# Interplay between *Yersinia pestis* and its flea vector in lipoate metabolism

**DOI:** 10.1038/s41396-020-00839-0

**Published:** 2021-01-21

**Authors:** Typhanie Bouvenot, Amélie Dewitte, Nadia Bennaceur, Elizabeth Pradel, François Pierre, Sébastien Bontemps-Gallo, Florent Sebbane

**Affiliations:** Univ. Lille, Inserm, CNRS, CHU Lille, Institut Pasteur de Lille, U1019 - UMR 9017 – CIIL - Center for Infection and Immunity of Lille, F-59000 Lille, France

**Keywords:** Bacterial pathogenesis, Metabolism

## Abstract

To thrive, vector-borne pathogens must survive in the vector’s gut. How these pathogens successfully exploit this environment in time and space has not been extensively characterized. Using *Yersinia pestis* (the plague bacillus) and its flea vector, we developed a bioluminescence-based approach and employed it to investigate the mechanisms of pathogenesis at an unprecedented level of detail. Remarkably, lipoylation of metabolic enzymes, via the biosynthesis and salvage of lipoate, increases the *Y. pestis* transmission rate by fleas. Interestingly, the salvage pathway’s lipoate/octanoate ligase LplA enhances the first step in lipoate biosynthesis during foregut colonization but not during midgut colonization. Lastly, *Y. pestis* primarily uses lipoate provided by digestive proteolysis (presumably as lipoyl peptides) rather than free lipoate in blood, which is quickly depleted by the vector. Thus, spatial and temporal factors dictate the bacterium’s lipoylation strategies during an infection, and replenishment of lipoate by digestive proteolysis in the vector might constitute an Achilles’ heel that is exploited by pathogens.

## Introduction

Multicellular organisms are a bonanza for those who know how to make the most of them, and pathogens, especially vector-borne pathogens, excel in this profit game [1–3]. However, to colonize the host, a pathogen must overcome or circumvent a variety of metabolic issues- even in the lumen of the host’s gut, where microbes are bathed in the nutrients provided by an ingested meal [4]. Indeed, the lumen content’s composition depends on the host’s diet and absorptive processes in the gut’s various compartments [3, 5, 6]. Furthermore, the gut lumen is an arena where pathogens compete with the microbiota for nutrient acquisition [7]. In other words, the availability of a nutrient varies throughout the process of gut colonization. It is therefore not surprising that many microorganisms have both a salvage and a biosynthetic pathway for a given nutrient, in order to survive in fluctuating and sometimes crowded gut environments [8, 9].

It is generally thought that when a nutrient is available in the environment, the microorganism prefers its salvage pathway to its biosynthetic pathway because the former is more cost-effective. Based on this assumption, one can intuitively deduce the source and origin of the nutrients scavenged by the pathogen during infection. Consequently, one can intuitively presume when and where the salvage and biosynthetic pathways for the nutrient of interest are respectively used during an infection. However, our knowledge is still fragmented with regard to (i) when and how the pathogen takes advantage of ingested nutrients, (ii) the exact source and origin of the nutrients used by the pathogen during an infection, and (iii) the spatial and temporal interplay between a pathogen and its host.

The plague agent, *Yersinia pestis*, is a Gram-negative bacterium that efficiently spreads through mammalian and flea hosts. In the flea, *Y. pestis* remains confined to the foregut (proventriculus) and midgut until it is transmitted to a new mammalian host (Fig. S1) [10]. Our knowledge of *Y. pestis*’ ability to detect, acquire and metabolize nutrients during flea infection is very limited [11–14]. The only proteins known to be involved in uptake and metabolic are the outer membrane porin OmpF, two ribose phosphate isomerases A (RpiAs) and the ribose phosphate epimerase (Rpe) [15, 16]. The nutrients imported by OmpF and the compounds synthesized by RpiAs and Rpe are important for colonization of the proventriculus but not for colonization of the midgut. In the foregut, these compounds are presumably involved in *Y. pestis*’ production of an extracellular biofilm that consolidates a soft bactericidal mass produced in the proventriculus and that entraps the bacilli (Fig. S1) [15, 17]. Ultimately, the consolidated mass causes freshly drawn blood to be blocked in the proventriculus, preventing meal ingestion in the midgut [10]. The direct consequences of blockage of the proventriculus include (i) the contamination and regurgitation into the dermis of the fresh blood drawn by the infected flea and (ii) an increase in the biting rate as the “blocked” flea starves to death [10, 11, 18]. Therefore, blockage boosts plague transmission.

The lack of knowledge on the “nutritional” mechanisms used by *Y. pestis* to infect fleas also applies to other aspects of blockage. At present, only a handful of loci are known to be necessary for the production of a successful infection in the vector and half of them are regulatory genes [15, 17, 19–27]. This is probably due to the difficultly of performing studies in arthropod vectors. Today’s methods require specific skills and are time consuming—making them unsuitable for the high-throughput screening of libraries of mutant microorganisms in the arthropod infection model. Furthermore, the flea has been neglected in the study of vector-borne diseases, even though it transmits pathogenic viruses, bacteria, and parasites of importance in veterinary and human health [28].

Here, we first describe our development and application of a new method for quickly and easily screening an unprecedented number of *Y. pestis* mutants in the flea. We then describe an in-depth study of a selected mutant that provided insights into the bacterial mechanisms leading to flea blockage. One of these mechanisms is the salvage and biosynthesis of lipoate—an essential enzyme co-factor. Interestingly, the salvage pathway’s lipoate/octanoate ligase LplA is an important factor in the biosynthesis of lipoate in the proventriculus but not in the midgut. Counterintuitively, we also found that *Y. pestis* does not use the free lipoate contained in the blood because this compound is rapidly depleted by the flea. Instead, *Y. pestis* uses the pool of lipoate replenished by digestive proteolysis—presumably in the form of lipoylated peptides. Taken as a whole, our data highlight (i) the spatial relationships between the lipoate pathway enzymes, and (ii) the importance of bacillus-vector temporal interplay in bacterial lipoate metabolism (the source of lipoate). Lastly, we suggest that the depletion of specific nutrients (such as lipoate) is a defense mechanism against infection and digestive proteolysis constitute weakness exploited by pathogens during an infection.

## Materials and methods

### Strains and plasmids

The bacterial strains and plasmids used in the study and their characteristics are listed in Table S1. *Escherichia coli* DH5α and *Y. pestis* KIM6 + were used respectively to clone sequences of interest, and to identify and study the various genes’ roles in flea blockage. The *Xenopsylla cheopis* rat flea was reared at the Institut Pasteur de Lille.

### In vitro bacterial growth

Lysogeny broth (LB) and Brain Heart Infusion (BHI; Becton Dickinson, France), M9-based medium (24 mM Na_2_HPO_4_ 7H_2_O, 11.02 mM KH_2_PO_4_, 4.28 mM NaCl, 9.35 mM NH_4_Cl, 0.66 μM FeSO_4_, 0.4 mM MgSO_4_, 0.2 mM CaCl_2_, 0.5% thiamin, 0.1 M succinate and 2.7 mg/mL vitamin-free casamino acids [Merck]), heparinized blood, plasma, and intact and lysed blood cells from OF-1 female mice (Charles River) were used for in vitro growth experiments. As necessary, media were supplemented with kanamycin (50 μg/mL), trimethoprim (25 μg/mL), ampicillin (100 μg/mL) or zeocin (50 μg/mL). Furthermore, LB was supplemented with acetate (5 mM), succinate (5 mM) and glycine (5 mM) to select mutants with defects in the lipoate salvage and/or biosynthesis pathways. M9-based medium and whole blood were supplemented with 8-bromooctanoate (1–8 µg/mL; Merck) or lipoate (48 mM; Merck) when required. Lastly, bacteria were also cultured in gut content from female fleas collected at different time points after feeding. In some experiments, the gut content was incubated with proteinase K (20 mg/mL, Merck) for 4 h at 25 °C. To generate “flea gut content medium”, intact guts were collected from 20 fleas in phosphate buffered saline (PBS) under a binocular microscope. Each gut was carefully washed in PBS, transferred in a microtube containing 60 µl of PBS, gently pierced (to release the content prior), and removed. Lastly, the flea gut content medium was generated by combining 10 µL from the microtube with 90 µL of PBS. However, when the gut contents were treated with proteinase K, 40 flea guts were collected in 100 µL of PBS. After homogenization, 50 µl of the homogenate were transferred into two distinct tubes. Lastly, 10 µL of PBS containing proteinase K (or not, as control) were added.

To compare the growth rate of different *Y. pestis* strains, bacteria cultured in LB at 28 °C were centrifuged, washed three times in PBS, and then suspended in PBS to give a final inoculum of 5 × 10^4^ bacteria/mL. After inoculation, bacteria were cultured at 21 °C with shaking. The growth in M9, LB, and BHI was monitored by measuring the OD_600nm_, whereas growth in blood and its derivatives was measured by counting the colony-forming units (CFUs), as described previously [12]. Lastly, growth in flea gut content was determined by measuring bioluminescence [29].

### Flea infection

Starved fleas were allowed to feed for 1 h on heparinized mouse blood supplemented (or not) with 8-Bro (8 µg/mL) and contaminated with 5 ×10^8^ bioluminescent or nonbioluminescent *Y. pestis*/mL, as described previously [16]. To measure the brightness, fed fleas were anaesthetized at 4 °C to distribute a cohort of males, a cohort of females or an equal number of males and females into the wells of a 96-well white plate (1 flea per well; Greiner) on ice. The plate was sealed with a transparent plastic film (ThinSeal^TM^), loaded into a dark box for 10 min, and then transferred to the Centro XS³ LB 960 luminometer (Berthold) for 5 min prior to signal acquisition (to reduce background noise and allow the fleas and the plate to warm up). The signal was acquired for 5 s (on the day of infection) or 15 s (on the following days). After each measurement, fleas were pooled in a cage and then housed in a climatic chamber (75% humidity, 21 °C) until the next measurement. Fleas displaying a RLU above the background level (determined to be 2.6 Log_10_ RLU) were defined as positive (i.e., infected).

To determine the blockage rate (defined as the presence of fresh red blood in the flea’s foregut but not in the midgut), cohorts of fed fleas (with equal numbers of males and females) were collected and analyzed twice a week for a 4-week period, as described previously [16]. The time course of gut colonization was studied by counting the CFUs grown on LB agar plates containing 1 µg/mL Irgasan and 10 µg/mL hemin and incubated at 28 °C for 48 h after an individual triturated female flea had been spread on it, as described previously [16]. However, when infected fleas were fed artificially with blood supplemented with 8-Bro (i.e., 2 days post infection), fleas were distributed across the wells of a 96-well white plate (1 flea per well). The plate was sealed with a transparent plastic film and fleas were monitored for defecation every 6 h for the following 24 h. Fleas that defecated were collected immediately after defecation, and the bacterial load was measured. We used this approach because defecation is the only reliable means of knowing whether a flea has had a meal two days after the previous meal—unlike the starved, hungry flea used for infection.

To monitor the strains’ ability to colonize the proventriculus, female fleas were infected with *Y. pestis* expressing the green fluorescent protein (GFP) from the pAcGFP plasmid (Addgene). Fluorescence photos of the proventriculus of fleas selected at random were taken with the Eclipse CiS fluorescence microscope (Nikon) mounted with a B-2A emission filter (Nikon) and a Sight DS-F1c camera (Nikon). The photos were then processed (using ImageJ software) to measure the surface area within the proventriculus (yellow) occupied by bacteria (green) [15].

### Genetic engineering

The plasmids and primers used are listed in Tables S1 and S2. We generated a *Y. pestis* strain (Yp^lux*^) harboring a promoter-free *luxCDABE* operon at the att Tn*7* site, in order to evaluate various promoters controlling the expression of the *lux* operon. The strain was built as follows. First, we inserted the miniTn*7*-*aphA*-*P*_*tolC*_::lux at the att Tn*7* site by co-transforming *Y. pestis* with pLOU034 and pTNS2 [29, 30]. Next, we (i) deleted the *aphA* antibiotic resistance cassette flanked by flippase recognition target (FRT) sites using the pFLP2 plasmid [30], (ii) cured the pFLP2 by streaking bacteria on plates of LB agar supplemented with 5% sucrose, and (iii) replaced the *tolC* promoter with a kanamycin resistance cassette by applying the lambda Red recombinase system and the plasmids and primer sets listed in the Supplementary Tables [16]. Lastly, the kanamycin-resistant cassette (flanked by the target sequence of the I-*Sce*I endonuclease) was replaced by a promoter of interest (*P*_*glnB*_, *P*_*nlpD*_, *P*_*pldB*_, *P*_*yjeH*_ or *P*_*ychJ*_) by combining the lambda Red recombinase system with the I-*Sce*I selection method as described previously [16], and using the plasmids and primer sets listed in the Supplementary Tables. Each clone was plasmid-cured by streaking on LB agar plus 5% sucrose.

We also generated a bioluminescent *Y. pestis* strain (Yp^lux^) lacking antibiotic resistance and harboring the *P*_*cysZK*_*-luxCDABE* construct at the att Tn*7* site, in order to evaluate mutants lacking *Y. pestis* genes in vivo and in vitro. This strain was generated using the same strategy as for Yp^lux*^, excepted that pLOU037 was used instead of pLOU034.

*Y. pestis* mutants lacking one or more of the genes previously reported as being activated during infection in the flea and *E. coli* mutants were generated using the lambda Red recombinase system, as described previously [12, 16, 31]. Briefly, the mutation was generated by replacing the sequence of interest with a selective marker (that had been amplified from the vectors listed in Table S1) then checked in a PCR assay (using the primer sets shown in Table S2). For complementation, the sequence of interest was amplified by PCR using the primer sets given in Table S2, and cloned into *E. coli* DH5α using the TA cloning kit with pCRII or pCR2.1 and pCR Blunt (ThermoFisher Scientific). The cloned sequences were checked by sequencing prior to electroporation into *Y. pestis*.

### Immunoblotting

Overnight cultures of bacteria grown in LB at 28 °C were harvested by centrifugation, washed twice in PBS, and lysed using the FastPrep instrument and lysing matrix B tubes (MP Biomedicals). After centrifugation, the supernatant was collected and the total protein was assayed with a Pierce BCA Protein Assay kit (Thermo Scientific). Equal amounts of proteins for testing were separated by SDS-PAGE. Two gels were run at the same time: one was stained with Coomassie blue dye (to control the loading) and the other gel was used for electrophoretic transfer onto a nitrocellulose membrane. The membrane was reversibly stained with Ponceau S stain (to check for correct protein transfer) and then incubated with primary polyclonal anti-lipoate antibody and a secondary anti-rabbit IgG conjugated to horseradish peroxidase (both from Merck). The chemiluminescence of the immunoreactive proteins was visualized and quantified using the LAS-3000 apparatus and Multi-Gauge software, respectively (both from Fujifilm).

### In vitro and in vivo lipoic acid assays

The amount of lipoate in the various media and in vivo was assayed using Hebert and Guest’s method, as previously described [32] with some modifications. This assay is based on a comparison of *Y. pestis* Δ*lipA’*s ability to grow in a medium of unknown lipoate concentration with its ability to grow in M9-based medium (whose composition is detailed above) supplemented with various known concentrations of lipoate (i.e., a standard curve). More specifically, *Y. pestis* Δ*lipA* grown overnight in LB at 28 °C was centrifuged, washed three times in PBS, and suspended in PBS prior to inoculation of the medium of interest with 5.10^4^ bacteria/mL final. After incubation at 28 °C with shaking for 22 h, serial dilutions of the medium were plated on LB agar, and CFUs were counted after a 48-h incubation at 28 °C. To determine the quantity of lipoate in vivo, the flea gut was collected, washed in PBS, and perforated to release the content into 5 µL of PBS. This volume was added to M9-based medium, which was then inoculated with *Y. pestis* Δ*lipA* to determine the amount of lipoate.

### Blood cell content in the gut of fleas

The digestive tracts of fed female fleas (*n*  =  5 per time point) collected immediately and then 6, 12, 24, 30, 36, 48  h after feeding were perforated to release their contents into 5 µL of PBS. The samples were placed immediately between a slide and coverslip and analyzed under a microscope for the presence or absence of blood cells.

## Results

### A new in vivo method for rapid identifying *Y. pestis* genes required for flea blockage

**Fig. 1:**
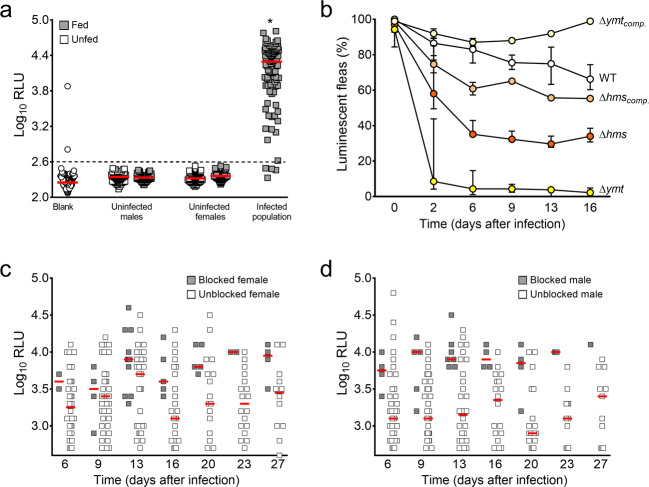
Luminescence readings from fleas fed on sterile blood or blood contaminated with WT, Δ*ymt* or Δ*hmsHFRS Y. pestis* mutants. **a** Well brightness (log_10_ relative light units (RLUs)) on a 96-well plate (white circles), either empty (blank) or containing either a single starved male or female flea (white squares) or a single male or female flea collected immediately after feeding on sterile blood or blood contaminated with a bioluminescent *Y. pestis* strain harboring the *P*_*cysZK*_-*luxCDABE* at the att Tn*7* site (grey squares). The red bars represent the median values. Blank: *n* = 192; uninfected fleas: *n* = 95–96; infected population, *n* = 96 fleas (sex ratio: 1:1). *p < 0.0001 in a one-way analysis of variance with Dunnett’s correction for multiple comparisons. The dashed line indicates the luminescence value (2.6 log10 RLU) above which a flea was considered to emit a signal over the background. Selection of this value reduced the likelihood of false positives and kept the false-negative rate relatively low. **b** The proportion of luminescent, live fleas at different time points (in days) after a blood meal contaminated with WT, Δ*ymt* or Δ*hmsHFRS Y. pestis* strains harboring P_*cysZK*_*-luxCDABE* at the att Tn*7* site and complemented (comp.) or not with a wild-type copy of *ymt* or *hmsHFRS* on a plasmid. A cohort of 96 fleas (sex ratio: 1:1) was sampled after infection. The data correspond to the cumulative results for 2 to 5 independent infections (WT), 5 infections (Δ*hmsHFRS*), 4 infections (Δ*ymt*), and 2 infections (complemented strains). However, data from one of the experiments were not collected after day 6. Hence, data shown >6 days post-infection correspond to the cumulative results for 1 to 4 independent infections. **c**, **d** Brightness of live female and male fleas considered to be blocked (grey squares) or not (white squares), over a 27-day period after infection with a WT bioluminescent strain. Each symbol represents the value obtained for a single, live insect. Fifty-nine females and 61 males were collected after infection.

We sought to develop a means of circumventing the following obstacle: the flea infection model currently used to identify *Y. pestis* genes required for blockage (by screening libraries of *Y. pestis* mutants) is unsuited to high-throughput screening. To this end, we took advantage of the properties of the *luxCDABE* operon from *Photorhabdus luminescens* because it confers bacteria with the ability to produce light autonomously and has been used as a bioreporter for monitoring bacteria in live animal models of infection [29, 33–35]. We first attempted to generate a *Y. pestis* strain with the brightest, most innocuous bioreporter construct, so that it could be used to identify the bacterial genes needed for flea blockage. We notably produced and evaluated six strains harboring *luxCDABE* at the att Tn*7* chromosomal site, according to the procedure described in the Methods section. The operon was under the control of a promoter whose strength was expected to range from high to low (*P*_*cysZK*_*, P*_*glnB*_, *P*_*nlpD*_, *P*_*pldB*_, *P*_*yjeH*_*,* or *P*_*ychJ*_*)* [13, 29]. When we determined the brightness of cohorts of fleas (each of which was infected with a single bioluminescent strain), only the strain harboring the *P*_*cysZK*_-*luxCDABE* construct was luminous enough to be detected (using a luminometer) in >10% of infected living fleas both immediately and several days after infection. In fact, more than 95% of male and female fleas fed on blood contaminated with this luminescent *Y. pestis* strain were bright enough to be detected immediately after infection (Fig. 1a). Furthermore, ~65% of live, infected fleas were still luminous 16 and even 27 days after the infected meal (Fig. 1b, white circles). Importantly, this strain blocked fleas at a rate similar to that of its parental strain (32% vs. 33%, respectively). Thus, using bioluminescence, we were able to track *Y. pestis* in living fleas for a long period without adversely affecting the bacteria’s ability to infect flea. Despite this advantage, blocked male and female fleas (which presumably contained more bacteria than an unblocked flea) were not always brighter than unblocked fleas (Fig. 1c, d); hence, we could not rely on the luminescence reading alone to identify genes of importance in flea blockage. However, a flea can clear an infection more easily when a *Y. pestis* mutant lacks a locus needed for flea colonization (i.e., *ymt*) or for production of the proventriculus-blocking biofilm (i.e., *hmsHFRS*) [17, 25]. Hence, we hypothesized that in fleas having ingested a bioluminescent *Y. pestis* strain, the change over time in the proportion of luminescent, live insects would indicate which mutants were unable to produce a transmissible infection. Consistently, almost no fleas infected with a bioluminescent Δ*ymt* mutant were luminescent between 2 days and 2 weeks post infection (Fig. 1b). In contrast, ~40% of fleas infected with the bioluminescent Δ*hms* strain remained bright from 6 to 16 days post-infection (Fig. 1b). However, this percentage was below that calculated for the wild-type strain and the complemented Δ*hms* and Δ*ymt* mutant strains. We concluded that bioluminescence measurement is a valuable approach for identifying *Y. pestis* mutants with defective infection in the flea. Furthermore, this approach is around four times faster than the current method, and requires fewer specialist skills [16].

### Resistance to oxidative stress, antimicrobial peptides, and osmotic stress characterizes *Y. pestis*’ response during flea infection

**Fig. 2:**
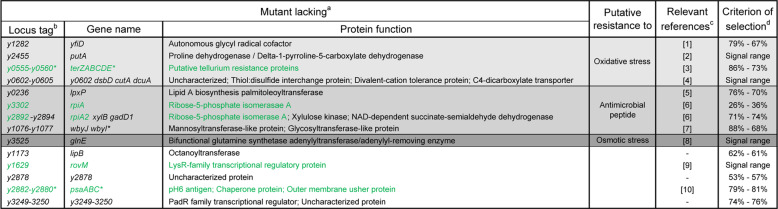
List of bioluminescent *Y. pestis* mutants with impaired flea infection. **a** The symbol “-“ (e.g., in “y0555-y0560”) means “to”; **b** genes highlighted in green but lacking the green symbol “*” were previously identified as being required for flea infection. Genes highlighted in green and bearing the green symbol “*” are known to be activated by a regulatory system that is important in flea infection. **c** The relevant references are given in the supplementary material. **d** Figure S2 shows the data on the selected mutants by signal range.

Our bioluminescence-based approach paved the way for the detection of defective mutants in the flea infection model at an unprecedented rate. Hence, we next generated and individually screened mutants lacking one or more of the genes previously reported as being activated during infection in the flea; a total of 164 were tested [13]. In view of the data in Fig. 1b and the many experiments performed, we considered that a mutant was defective when the percentage of luminescent fleas 2 and 9 days post-infection was <80% and/or 75%, respectively. However, we fortuitously noticed another type of mutant that might be of interest: the range of RLU values of infected fleas on day 9 post infection was narrower, and most insects emitted a much weaker signal (albeit above the background) than wild-type-like strains did. The mutants selected with the two above-mentioned criteria were then retested to confirm or refute their deficiency. After this second round of screening, 14 mutants were found to have a reproducible defect (Figs. 2 and S2). Three of these 14 mutants lacked genes thought to be important in flea infection, such as *rovM*—a gene whose role had previously only been revealed by its competitive index in a mixed infection with a WT strain [23]. Two other strains lacked a locus (*psaABC* or *y0555-y0560*) activated in the flea by a two-component regulatory system (PhoP-PhoQ) that is important for flea blockage [36]. Lastly, from a biological viewpoint, 65% of the identified mutants lacked genes previously reported (in *Y. pestis* or other bacterial species) as being necessary for in vitro resistance to oxidative stress, antimicrobial peptides, or osmotic stress (Fig. 2). In conclusion, the screening data (i) validated our method for the rapid identification of genes required for flea infection, (ii) provided insights into the stresses encountered by *Y. pestis* during flea infection, and (iii) identified several potentially new genes—including uncharacterized ones—required for the various steps leading to flea blockage or required for optimized flea colonization (i.e., fitness).

### The *y1173* and *y1171* genes encode a lipoate biosynthetic pathway of importance for flea blockage

**Fig. 3:**
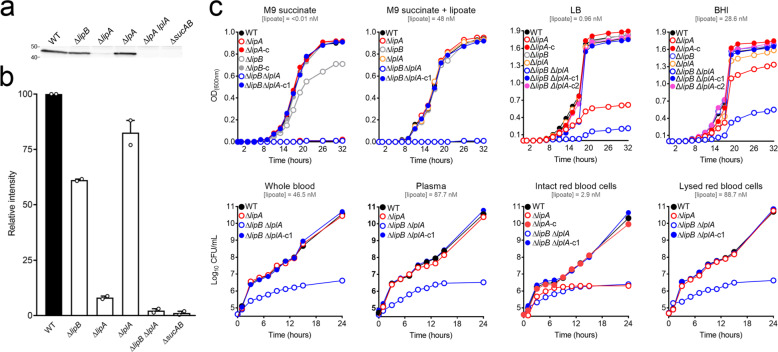
Analysis of lipoylation in and growth of WT *Y. pestis* and its isogenic mutant strains lacking *y1173* (*lipB*), *y1171* (*lipA*) and/or *y1926* (*lplA*). **a** An immunoblot (using a polyclonal, lipoate-specific antibody) of whole-cell lysate of WT and mutant *Y. pestis* strains grown overnight at 21 °C with shaking in lysogeny broth (LB). Each well was loaded with the same amount of protein. The mutant Δ*sucAB* was used to locate the presence of the known lipoylated protein SucB. **b** The bars show the relative intensity of the bands present on the immunoblot. The relative intensity was calculated by dividing the normalized intensity of the band of interest by that of the WT strain. The mean (standard error of the mean (SEM)) of 2 independent blots is shown. **c** WT and mutant strains washed in the medium of interest (to reduce the carry-over of nutritional supplements) were cultured at 21 °C with shaking in different media. Growth was monitored as (i) the optical density (OD_600nm_) when cultured in M9 succinate supplemented with (in some cases) lipoate, LB or brain heart infusion (BHI), and (ii) the CFU count when incubated in whole blood, plasma, or intact or lysed isolated blood cells. The concentration of lipoate present in each medium was determined and given. “–c” indicates a mutant complemented with a WT copy of the deleted gene; “–c1” and “–c2” indicate the presence of a WT copy of *lplA* and *lipB*, respectively. The results of a representative growth curve from two independent experiments are shown.

**Fig. 4:**
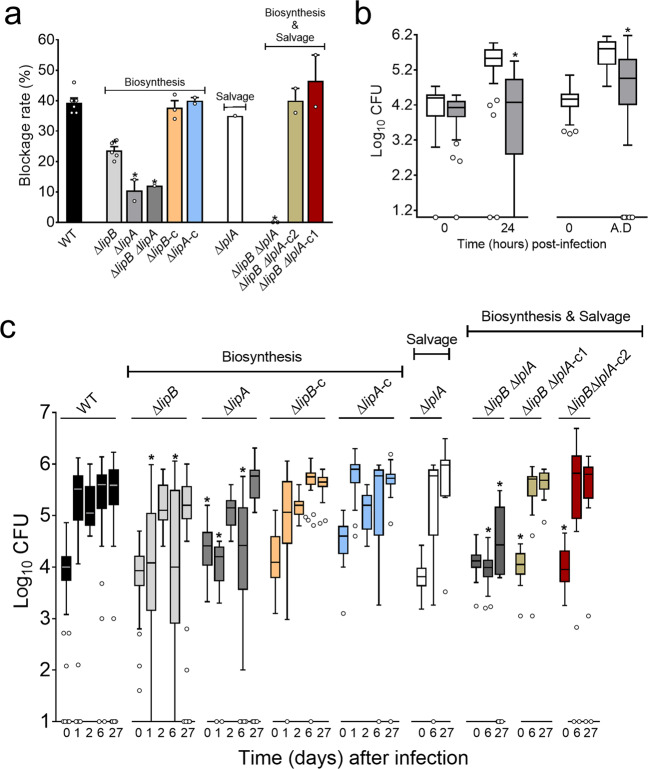
Flea blockage and bacterial growth in fleas fed on blood contaminated with a WT *Y. pestis* strain or a mutant strain lacking *lipB*, *lipA* and/or *lplA*, and supplemented or not with the lipoate analogue 8-bromooctanoate. **a** The flea blockage rate during the 4 weeks following infection by the strain of interest. The mean and SEM of 2 to 6 independent infections are shown, except for the Δ*lipA* Δ*lipB* and Δ*lplA* mutants (data from one infection). “–c” indicates a mutant complemented with a WT copy of the deleted gene; “–c1” and “–c2” indicate the presence of a WT copy of *lplA* and *lipB*, respectively. *: a significant difference *vs*. the WT strain (*p* < 0.0003 in a one-way analysis of variance with Dunnett’s correction for multiple comparisons). **b** “In the left panel, box-and-whisker (Tukey) plots represent the number of *Y. pestis* (Log_10_ CFUs) in fleas fed on contaminated blood supplemented (grey) or not (white) with 8-bromooctanoate (8-Bro; 8 µg/mL) and collected immediately after and 24 h after the infection. In the right panel, box-and-whisker (Tukey) plots represent the bacterial load (Log_10_ CFUs) in fleas fed on blood containing *Y. pestis* (but not Bro) and collected immediately (0) after infection. The plots also represent the number of *Y. pestis* in fleas that had defecated (A.D.) following a sterile blood meal 2 days post-infection and supplemented (grey) or not (white) with 8-Bro (8 µg/mL). The data correspond to the cumulative results of two independent infections, and up to 20 fleas were used for each time point (i.e., a total of 33–40 fleas). *p < 0.0001 compared with the WT control in a one-way analysis of variance with Dunnett’s correction for multiple comparisons. **c** Box-and-whisker (Tukey) plots of the bacterial load (Log_10_ CFUs) in fleas fed on blood contaminated with the strain of interest. The data correspond to the cumulative results of 2–5 independent infections in which 19 to 20 fleas were randomly collected on different days post-infection (i.e., a total of 39 to 120 fleas). However, the data on D2 for the WT and Δ*lipB* strains and at all tested days for the Δ*lipA* and complemented strains result from one infection. “–c” indicates a mutant complemented with a WT copy of the deleted gene; “–c1” and “–c2” indicate the presence of a WT copy of *lplA* and *lipB*, respectively. *: a significant difference vs. the WT strain (*p* < 0.05 in a one-way analysis of variance with Dunnett’s correction for multiple comparisons).

We identified the *y1173* gene as being required for flea infection. The gene databases suggest that *y1173* encodes the octanoyltransferase LipB (sharing 61% identity with LipB from *E. coli*), which catalyzes the first step in the synthesis of lipoate—an essential cofactor covalently attached to enzymes in central metabolism (e.g., succinate dehydrogenase (SucB)) in all kingdoms [37–41]. Notably, LipB covalently attaches an octanoyl group to acceptor proteins. Next, two sulfur atoms are subsequently inserted into the octanoyl group by the lipoate synthase A (LipA) to form the lipoate cofactor. LipA might be encoded by *y1171* in *Y. pestis*, and shares 92% identity with LipA from *E. coli* (Fig. S3) [37]. In line with these predictions, lipoylation of the SucB E2 subunit is abnormally low when a strain lacking *lipB* or *lipA* is grown in a rich medium (Fig. 3a). Furthermore, when the bacteria are cultured in M9 succinate medium, the lack of *lipA* induces auxotrophy for lipoate and the lack of *lipB* leads to an abnormally low growth rate, as reported for *E. coli* [40, 42] (see Fig. 3c for M9 succinate *vs*. M9 succinate + lipoate). Furthermore, the growth impairment of the Δ*lipA* and Δ*lipB E. coli* mutants was no longer present when the mutants respectively expressed *lipA* and *lipB* from *Y. pestis* (Fig. S4). Lastly, we found that a nonbioluminescent *Y. pestis* lacking *lipA* and/or *lipB* was less able to block fleas, relative to the WT strain (Fig. 4a). Thus, *Y. pestis* has a lipoate biosynthetic pathway encoded by *lipA* and *lipB*, and the bacterium uses this pathway to block fleas effectively.

### The *y1926* gene encodes a lipoate/octanoate ligase LplA involved in flea blockage

Although the lipoate biosynthetic pathway is important for the production of a transmissible infection in fleas, it is not essential (Fig. 4a). A Δ*lipB* mutant blocked 50% more fleas than a Δ*lipA* mutant—indicating that octanoylation by LipB is less important than lipoate synthesis by LipA. This finding is in line with the lipoylation profile for bacteria grown in vitro (Fig. 3a, b). Indeed, the deletion of *lipA* or *lipB* reduced but did not abolish lipoylation, and the deletion of *lipB* had less impact than the deletion of *lipA*. Taken as a whole, these data indicate the presence of a redundant lipoylation and octanoylation pathway. Consistently, the growth rate of *Y. pestis* deleted for Δ*lipA* was positively correlated with the concentration of lipoate present in the culture medium (M9 succinate< LB < BHI)—suggesting that a lipoate salvage pathway rescues the absence of a biosynthetic pathway (Fig. 3c). Furthermore, gene databases predicted that *Y. pestis y1926* encodes LplA, which attaches octanoate [43] and lipoate [44] scavenged from the environment to apoenzymes. LplA from *Y. pestis* shares 75% identity with LplA from *E. coli*. In agreement with these predictions, the additional deletion of *y1926* in a *Y. pestis* strain lacking the lipoate biosynthetic pathway (Δ*lipB*) abolished growth in M9 succinate supplemented with lipoate (Fig. 3c) and the lipoylation of SucB in rich media (Fig. 3a). Thus, *y1926* appears to code for the salvage pathway’s LplA. Comparisons of the growth curves and lipoylation profiles for strains lacking (or not) *lipA*, *lipB* and/or *lplA* cultured in rich media (LB and BHI) suggested that *Y. pestis* LplA has also an octanoate ligase activity (redundant with LipB’s activity) as well as lipoate ligase activity (Fig. 3). Indeed, the deletion of *lipB* or *lplA* did not induce poor growth (in contrast to the deletion of *lipA)*. Furthermore, the Δ*lipB* Δ*lplA* double mutant grew less efficiently than the single Δ*lipB*, Δ*lplA* and Δ*lipA* mutants or bacteria expressing a WT copy of *lipB* or *lplA*. Lastly, we found that a Δ*lipB* Δ*lplA* mutant cultured in M9 succinate lacking lipoate (i.e., in which LplA cannot act as a lipoate ligase) grew only when it expressed *lplA* (Fig. 3c; see the M9 succinate panel). This observation suggests that LplA has octanoate ligase activity. Given that LplA appeared to be involved in lipoate salvage and biosynthesis pathways, we next studied the putative gene product’s role in the flea. We sought to determine whether or not the presence of LplA explains (i) why the lipoate biosynthetic pathway is necessary but not crucial for flea blockage, and (ii) why octanoylation by LipB is less important than lipoate synthesis by LipA for flea blockage. We found that the Δ*lplA* Δ*lipB* double mutant was unable to block fleas, in contrast to the Δ*lplA*, Δ*lipB,* and Δ*lipA* single mutants (Fig. 4a). In other words, a mutant unable to perform octanoylation and lipoylation (the Δ*lplA* Δ*lipB* mutant) blocked fewer fleas than a mutant with a partial defect in octanoylation (Δ*lplA* or Δ*lipB* strain) or in lipoylation (Δ*lplA* and Δ*lipA*). Thus, the presence of *lplA* may explain the difference in blockage between strains lacking *lipA* and those strains lacking *lipB*.

Although our data suggested that the salvage pathway had a role in flea blockage, the disruption of this pathway alone was not associated with a low blockage rate (Fig. 4a). We therefore sought to determine whether the lipoate salvage pathway is active shortly after and/or long after infection. To this end, we took advantage of the lipoate analogue 8-bromooctanoate (BrO) known to inhibit LplA in various bacteria and parasites [43, 45–50]. As in other microorganisms, BrO inhibited the growth of LplA-producing *Y. pestis* strains (Fig. S5). Furthermore, BrO more effectively inhibited a mutant strain that relied more on the lipoate salvage pathway than on the biosynthetic pathway for optimal growth: 2 and 8 µg/mL of BrO inhibited the growth of the Δ*lipB* and WT strains, respectively. Although blood supplemented with 8 µg/mL BrO is toxic for *Y. pestis*, it is not for fleas—at least after two ingestions. Four days after the second feed, the mortality rate in both untreated and treated fleas (*n* = 100 in each group) was 2%. It was therefore possible to use BrO to determine whether *Y. pestis*’ salvage pathway is active in fleas. We found that the addition of BrO to the blood used to infect fleas or to sterile blood subsequently used to feed infected fleas was associated with a low number of *Y. pestis* recovered from the insects (Fig. 4b). Thus, the lipoate salvage pathway is active during flea infection.

In summary, our data suggest that two lipoate pathways are needed for flea blockage by *Y. pestis*: a salvage pathway and a biosynthetic pathway. The former depends on the lipoate ligase activity of LplA. The latter is a two-step process in which the octanoylation by the octanoyl transferase LipB and the octanoate ligase LplA is followed by sulfur transfer by the lipoate synthase LipA. Lastly, our data also indicate that lipoylation is not essential for flea infectivity, although it is crucial for flea blockage. Indeed, >90% of fleas that ingested *Y. pestis* lacking one or both lipoate pathways (i.e., the Δ*lipB*, Δ*lplA,* and Δ*lipB* Δ*lplA* strains) remained infected for 4 weeks post infection, which is similar to the data obtained with the wild-type strain (Fig. 4c).

### Although foregut and midgut colonization involves the lipoate biosynthesis and salvage pathways, the octanoate ligase activity of LplA is important only for foregut colonization

**Fig. 5:**
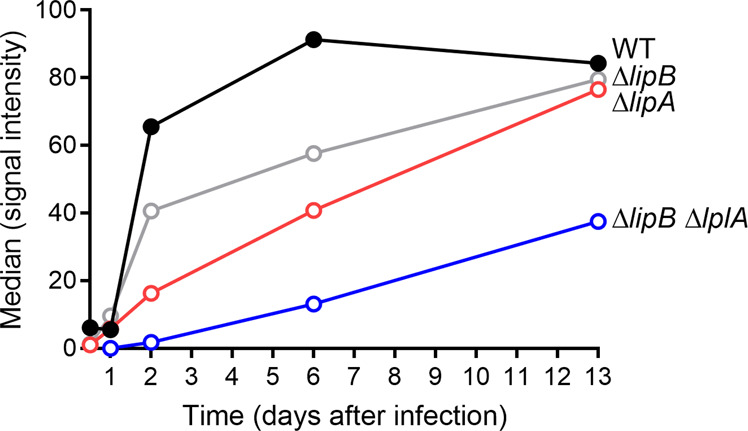
The time course of proventriculus colonization by WT, Δ*lipB*, Δ*lipA* or Δ*lipB* Δ*lplA Y. pestis* strains. Each circle represents the median fluorescence intensity from GFP-expressing bacteria present in the proventriculus of 20 *X. cheopis* fleas that had fed on blood infected with *Y. pestis* WT, Δ*lipB*, Δ*lipA* or Δ*lipB* Δ*lplA* and were collected at different time points post-infection (Fig. S6 shows the proventriculus, for determination of the median fluorescence intensity).

Although lypoylation is not important for flea infectivity, the Δ*lipB* Δ*lplA* count in fleas collected immediately after the meal and up to 4 weeks post-infection showed that the mutant was unable to heavily colonize the vector (Fig. 4c). This colonization profile contrasted with those obtained with the WT strain and mutants lacking one lipoate pathway or the other (i.e., the Δ*lipA*, Δ*lipB,* or Δ*lplA* strain). For the latter strains, the bacterial count increased over time and was similar 4 weeks post ingestion (Fig. 4c). Nonetheless, the Δ*lipA* or Δ*lipB* strains displayed a similar growth default in the first week post infection, which was not noticed with the Δ*lplA* mutant (Fig. 4c). In other words, *lplA* did not rescue the absence of *lipB* for colonization of the flea as a whole. This finding contrasts with the fact *lplA* appears to bypass partially the absence of *lipB* for flea blockage (i.e., foregut colonization or consolidation the proventricular mass) (Fig. 4a). Consistently, the loss of *lipB*, *lipA* or both *lipB* and *lplA* reduced *Y. pestis’* ability to colonize the proventriculus. However, the loss of *lipB* had less impact than that of *lipA*, and the double mutant Δ*lipB* Δ*lplA* was the most affected strain *(*Figs. 5 and S6). Thus, LplA’s octanoate ligase activity appeared to be involved at least for efficient foregut colonization but not for midgut colonization. Lastly, the importance of LplA’s octanoate ligase activity contrasts with the lesser importance of LplA’s lipoate ligase activity since a Δ*lipB* Δ*lplA* strain blocked and colonized fleas less well than a single Δ*lipB* or Δ*lplA* mutant (Fig. 4a, c). One can conclude that the lipoate ligase activity of LplA is involved for both foregut and midgut colonization. Altogether, our data prompted us to draw up a model in which *Y. pestis* synthesizes lipoate via LipA, LipB, and LplA (i.e., LplA’s octanoate activity) and uses environmental lipoate via LplA (i.e., LplA’s lipoate ligase activity) to effectively colonize and block fleas. However, lipoate biosynthesis pathway involves LipA, LipB, and LplA (i.e., LplA’s octanoate ligase activity) in the proventriculus but only LipA and LipB in the midgut. Hence, in contrast to midgut colonization, proventriculus colonization may involve the full array of enzymatic activities involved in lipoylation.

### Blood-derived lipoyl peptides (rather than free lipoate) may be a major source of lipoate for *Y. pestis* during flea infection

**Fig. 6:**
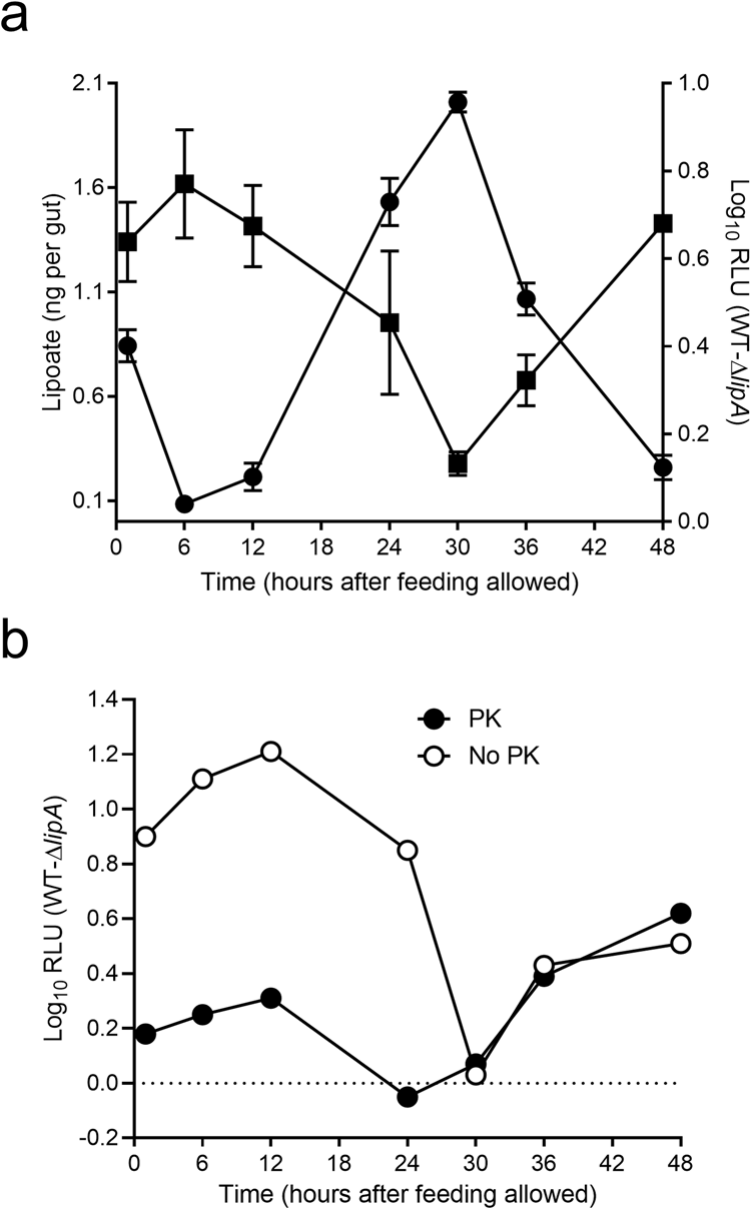
The relationship between the growth of *Y. pestis* requiring lipoate salvage, the amount of free lipoate, and digestive proteolysis in the flea gut. **a** The figures show the amount of lipoate in flea gut content collected at various time points after feeding (circles) and the growth of *Y. pestis* requiring lipoate salvage for growth in the corresponding gut content (squares). Growth efficiency was calculated by subtracting the difference in RLU counts measured for the WT strain and the mutant Δ*lipA* 24 h after their incubation ex vivo in flea gut content. For the amount of lipoate, the circles represent the mean ± SEM value of 5 independent samples. For the bacterial growth curve, the squares represent the mean ± SEM value of 5, 4, and 1 independent growth experiments (for the 1–24h time points, 30–36h time points, and 48 h time point, respectively). **b** Growth efficiency of *Y. pestis* requiring lipoate salvage for growth after incubation in flea gut content collected at various time points post-infection and treated (black circles) or not (white circles) with proteinase K. The circles represent the data from one experiment.

Strikingly, the growth curve of the Δ*lipA* and Δ*lipB* mutants in fleas had a sawtooth shape that contrasted with the curve of the WT strain (Fig. 4c). This presumably reflected the nutrient supply cycle for ingested blood. Whether this latter idea is true, lipoate supply is delayed after the meal because Δ*lip* mutants with a defect in lipoate synthesis only start to grow 24 h after ingestion whereas the WT strain starts to grow immediately after ingestion (Fig. 4c). This delay in nutrient acquisition is somewhat counterintuitive because only the loss of both salvage and biosynthetic pathways induced a growth defect for *Y. pestis* in blood (Fig. 4c). In other words, the *Y. pestis* lipoate auxotroph (the Δ*lipA* strain) grows normally in lipoate-rich blood (46 µg/mL) but is unable to use freshly ingested nutrients to grow in the flea gut. Thus, different sources of lipoate are used for growth before and after blood ingestion. We therefore conceived a model in which the flea quickly depletes nutrients from plasma, leaving bacteria in contact with intact blood cells and preventing them from accessing the nutrients they need to thrive. Next, the flea progressively lyses blood cells, which releases enough nutrients for bacterial growth. Lastly, the flea depletes the released nutrients, which again prevents bacterial growth. In line with this model, plasma and blood cell lysates (but not intact blood cells) supported effective growth of a Δ*lipA Y. pestis* strain because plasma and blood cell lysate contained sufficient amount of free lipoate for growth compared to intact blood cells (Fig. 3c). Furthermore, the concentration of lipoate in the flea gut dropped severely within 6 h of ingestion, increased steadily over the next 24 h (i.e., until 30 h post-feeding), and then fell again (Fig. 6a). Lastly, the curve describing the change over time in the amount of lipoate in the flea’s gut was the mirror image of the curve depicting the ability of the lipoate auxotrophic Δ*lipA* mutant (compared with its parental strain) to grow in the flea gut content collected at various times after blood ingestion (Fig. 6a). The mutant’s growth rate was positively correlated with the amount of lipoate available in the flea gut (R^2^ = 0.84) (Fig. S7). However, *X. cheopis* disrupts >99.5% of rat [51] and mouse blood cells within 6 h of ingestion (Fig. S8); these data contradict our model and thus indicate that free lipoate is not acquired by *Y. pestis*. Hence, we hypothesized that the source of lipoate acquired by *Y. pestis* in the flea gut are lipoylated proteins in blood cells and particularly lipoylated peptides released by digestive proteolysis. To test this hypothesis, we compared the ability of the Δ*lipA* mutant to grow in flea gut content collected at various times post-feeding and which was treated or not with proteinase K prior inoculation. We found that the proteolysis treatment alleviated the mutant’s growth defect (Fig. 6b). However, this alleviation did not occur after 24 h (presumably when the digestion has ended), which is consistent with the putative availability of blood-derived lipoyl peptides for growth. We conclude that the lipoate salvage pathway of *Y. pestis* during flea gut colonization relies on lipoylated peptides from mammal blood more than on free lipoate, and that digestive proteolysis is essential in this regard. Taken as a whole, our data highlight the contribution of *lplA* to the acquisition of the free and host-derived lipoate required for infection by *Y. pestis*.

## Discussion

Creating an inventory of the genes needed to produce an infection is the first step towards a better understanding of the complex mechanisms that underlie an infectious disease. To this end, in vitro models of putative in vivo conditions have been used to screen large number of mutants [52–57]. Although these methods are valuable, they only provide a narrow view of the living organism. This is why several techniques have been designed and used to screen mutant libraries in animal models [12, 57–61]. These methods often use a nonphysiological inoculum containing a very large number of different mutants, which may bias the conclusions. In other words, the screening of individual mutants in vivo remains the gold standard for identifying pathogenic factors. However, this large-scale screening is very burdensome. Our newly developed technique circumvents this technical bottleneck. In view of the flea’s small size and our ability to produce and handle hundreds of individuals, our model constitutes a unique opportunity for relatively high-throughput screening of a large number of mutants. To achieve this goal, we used a bioluminescence-based approach to track the change over time in the proportion of infected fleas (Fig. 1). Our approach was applied here to *Y. pestis* in fleas but could potentially be transposed to other flea-borne pathogens (such as *Rickettsia typhi* and *Trypanosoma lewisi* [62, 63]) or even pathogens transmitted by arthropods other than fleas—some of which are responsible for major human, veterinary and plant diseases.

Our approach generated the most complete survey yet of the genes required for flea infection. Our work almost doubled the list of genes though to have a role in flea infection by *Y. pestis* [16]. The predicted roles of the newly listed genes are in line with literature data indicating that the flea’s digestive tract is a hostile ecological niche due to the presence of reactive oxygen species, antimicrobial peptides, and fluctuations in osmolarity (Fig. 2) [15, 16, 64]. Thus, *Y. pestis* encounters the same stresses as other pathogens, and not only vector-borne pathogens, ingested by arthropods [65–68]. Hence, other arthropod pathogens might use the same strategies and genes used by *Y. pestis* to overcome the hostile arthropod gut environment. Since our study listed both characterized genes and uncharacterized conserved genes, it might open the door to the discovery of new, common mechanisms of infection.

Our subsequent investigation of the *lipB* gene showed that lipoylation (a post-translational modification of several central metabolic enzymes [37]) is essential for flea blockage but not for flea infectivity (Fig. 4a and c). Our results also suggested that levels of compounds synthesized by lipoylated enzymes (or their derivatives) or other nutrients present in the gut lumen are sufficient for infection but not for flea blockage. Therefore, lipoylation appears to be a means of boosting the pathogen’s metabolism, enhancing the infectious process and producing effective transmission. It will be of interest to determine whether the lypoylation-related blockage defect is merely the result of a metabolic defect slowing down bacterial growth. Indeed, lypoylation might also be involved (i) for optimal growth because it confers optimal resistance to a toxic compound and/or (ii) it allows the production of the molecules which enable the proventricular cast (produced very soon after the infection) to be firmly anchored. This “lipoylation boost” can be achieved in two ways: salvage and biosynthesis (Fig. 4). As in *E. coli* and other microorganisms, lipoate salvage in *Y. pestis* involves LplA and lipoate biosynthesis involves a two-step process initiated by LipB and terminated by LipA (Figs. 3 and S3) [39, 40, 43, 69–73]. However, LipB is not essential if LplA is present (Figs. 3 and 4). In addition to ligating scavenged lipoate to apoenzymes, LplA presumably also octanoylates apoenzymes. Indeed, this type of LplA activity has already been reported for *Staphylococcus aureus* and *E. coli* [42–44, 47, 74]. However, in vivo octanoylation by LplA was detected in *E. coli* only. In fact, the physiological role of octanoylation by LplA and its importance remained unknown, and some might consider it anecdotal. However, our data shed light on the physiological importance of LplA in lipoate biosynthesis and suggest that octanoylation by LplA is important for pathogenesis. Indeed, this enzyme activity appears to be required for optimal flea blockage (proventriculus colonization, at least) by *Y. pestis* but not for midgut colonization (Figs. 4 and 5). Thus, by boosting the initial step in lipoate biosynthesis, LplA might enable pathogens to overcome particular environmental obstacles. LplA can also boost lipoylation via its role in the lipoate salvage pathway, since the latter has a role in flea blockage and midgut colonization (Fig. 4). Thus, our data on LplA’s two activities and the two lipoate metabolic pathways emphasize the breadth and efficiency of lipoylation under appropriate environmental conditions. Overall, our findings (i) highlight the importance of the spatial relationship between the lipoate salvage and biosynthesis enzymes during an infection, (ii) expand our understanding of lipoic acid metabolism in pathogenesis, and (iii) outline an adaptive strategy that makes microorganisms extremely adept at lipoylation in a variety of ecological niches.

Among the ecological niches encountered by the majority of arthropod-borne pathogens, the gut lumen must always be overcome to produce an infectious cycle. Since arthropod-borne pathogens are acquired in a blood meal, it would be reasonable to suppose that they readily use the nutrients provided by the meal to develop. Our data repudiate this hypothesis—at least for lipoate. Although *Y. pestis* effectively uses free lipoate present in the serum and within mammalian blood cells to grow and—possibly—to enable transmission to the flea, the bacterium has more limited access to this source upon ingestion because the insect quickly depletes lipoate levels (Fig. 6). Due to rapid depletion, *Y. pestis* cannot take full advantage of the free lipoate contained in and then released from blood cells during the digestion process (Figs. 3 and 6). Thus, even if an ingested element is apparently readily available in the gut, microorganisms may not necessarily salvage before it is depleted by the host. It is therefore tempting to consider that the induction of deficiencies in essential compounds (such as lipoate) would be one of the host’s first lines of defense against potentially harmful ingested organisms in general and against exclusive lipoate scavengers (such as some Firmicutes and Apicomplexa, according to genome analyses) in particular [48, 75, 76].

**Fig. 7:**
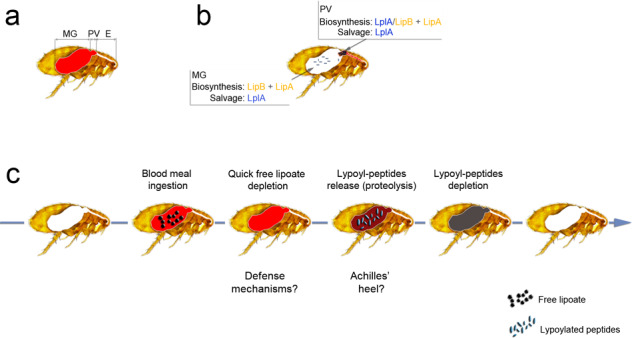
The ying and yang of blood meal digestion by the flea, and lipoate metabolism in *Y. pestis*. **a** During its ingestion with the blood, *Y. pestis* crosses the esophagus (E), the proventriculus (PV), and the midgut (MG). The bacterium remains confined in the MG and PV until transmission to a new host. **b** Spatial aspects of lipoate metabolism of *Y. pestis* in its flea vector. Lipoate biosynthesis and lipoate salvage from the flea gut lumen are not essential for flea infectivity but boost *Y. pestis*’ ability to produce a transmissible infection in its vector, i.e., the ability to block the fleas’ proventriculus (the brownish hatched mass). This blockage hastens the transmission of *Y. pestis* by the starving fleas during a feeding attempt. Lipoate biosynthesis is initiated by octanoylation and then lipoate synthesis by the octanoyl-transferase LipB and the lipoate synthase LipA, respectively. Octanolyation can be also catalyzed by LplA. However, LplA’s role in lipoate biosynthesis appears to be important in the proventriculus but not in the midgut. LplA also catalyzes the ligation of lipoate after salvaging the compound from the flea gut lumen. **c** Blood meal digestion by the flea. During the process of digestion, the fresh ingested blood meal (bright red) becomes progressively darker. The flea also quickly depletes free lipoate (black pentagons) contained in plasma and released from the disruption of blood cells; this prevents *Y. pestis* from easily accessing this essential co-factor. Next, digestive proteolysis replenishes a pool of lipoate—presumably in the form of lipoyl peptides (blue ellipses)—that can be used by *Y. pestis*. Lastly, the flea depletes lypoyl peptides and defecates the unprocessed part of the meal. Thus, a nutrient that appears to be readily available is not. Rapid depletion of ingested nutrients could viewed as a mechanism reducing ability of some invaders to grow. However, digestive proteolysis (which reconstitutes the rapidly depleted nutrients in a different form) might be constitute a weak point exploited by certain microorganisms to maintain themselves in the digestive tract and thus produce an infection.

Obviously, some organisms have evolved or acquired biosynthesis mechanisms to counteract nutrient deficiencies. However, biosynthesis is costly. In this regard, it is noteworthy that *Listeria monocytogenes* uses host lipoyl peptides (rather than free lipoate or synthesized lipoate) as a source of lipoate for survival inside macrophages and thus for virulence in mice [77, 78]. However, this bacterium uses free lipoate efficiently in vitro [77, 78]. *Staphylococcus aureus*, whose virulence is lipoylation-dependent, is able to use both free lipoate and host lipoyl peptides to lipoylate its enzymes [74, 79, 80]. Thus, when the level of free lipoate is low, the use of host-derived lipoyl peptides might be a cost-effective alternative to biosynthesis. Our present data suggest that *Y. pestis* acquires lipoate from host-derived peptides upon digestive proteolysis of blood, which occurs after free lipoate depletion (Fig. 6). Hence, assuming that rapid depletion of ingested nutrients is a defense mechanism against ingested microorganisms, one can further hypothesize that digestive proteolysis is an Achilles’ heel exploited by pathogens to indirectly scavenge the initially depleted nutrients. Lastly, our findings suggest that the sources of nutrients used by transiently or permanently pathogenic microorganisms in the host or vector’s gut are not always the most intuitive ones. It is important to bear in mind the role of digestion in a microorganism’s nutrient supply mechanisms (Fig. 7).

If lipoyl peptides are indeed a source of lipoate, one can hypothesize that lipoamidase activity (i.e., the release of lipoate from lipoyl peptides) exists as a side effect of the proteolysis. This enzymatic activity has been reported in several bacteria and in different mammalian sources, including serum [75]. Therefore, lipoamidase activity might occur in *Y. pestis* or the flea gut during digestion. Future experiments will be needed to identify the source of this activity and the factors involved in the hydrolysis of lipoyl peptides.

## Supplementary information

SUPPLEMENTAL

## Data Availability

All data are contained within the manuscript and/or Supporting Information files.
