# Supplementary material for: Interplay between Yersinia pestis and its flea vector in lipoate metabolism
Source: ISME J. 2021 Jan 21;15(4):1136–49. doi: 10.1038/s41396-020-00839-0 (PMC8182812; doi:10.1038/s41396-020-00839-0)
Supplement: Supplementary file 1 — SUPPLEMENTAL [file 41396_2020_839_MOESM1_ESM.pdf]

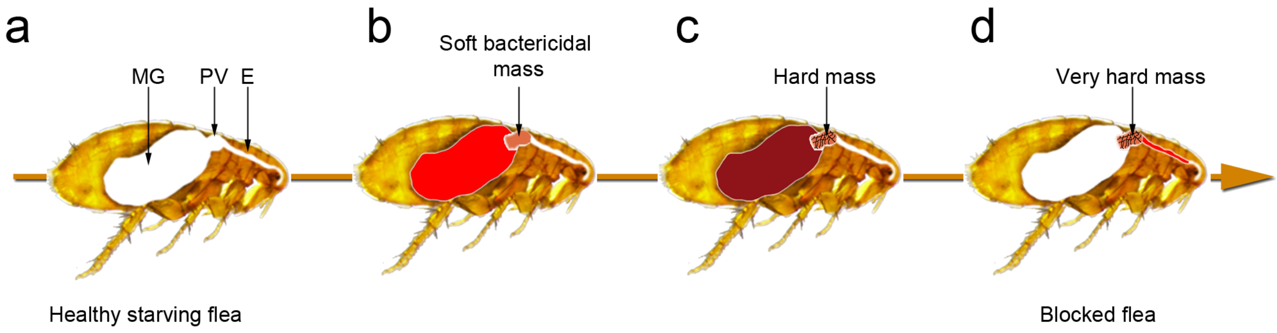

Figure S1. A simplified model of the steps leading to flea blockage. When a hungry or starved flea (a) takes a blood meal (bright red) containing *Y. pestis*, a soft bactericidal mass entrapping *Y. pestis* is molded into the shape of the proventriculus (b). During the digestion process, the blood (dark red) becomes darker. *Y. pestis* resists toxic compounds, replicates within and consolidates the soft mass (c). However, the mass is recurrently dislodged by a blood meal and recolonized by *Y. pestis* (not shown, see reference [6]) until it becomes enough hard to remain firmly anchored to the proventriculus and thus definitively blocks it. Blockage of the proventriculus prevents the flea from ingesting a new blood meal (d). This blockage starves the flea and leads to the regurgitation of bacteria at the fleabite site. MG, midgut; PV, proventriculus; E, esophagus.

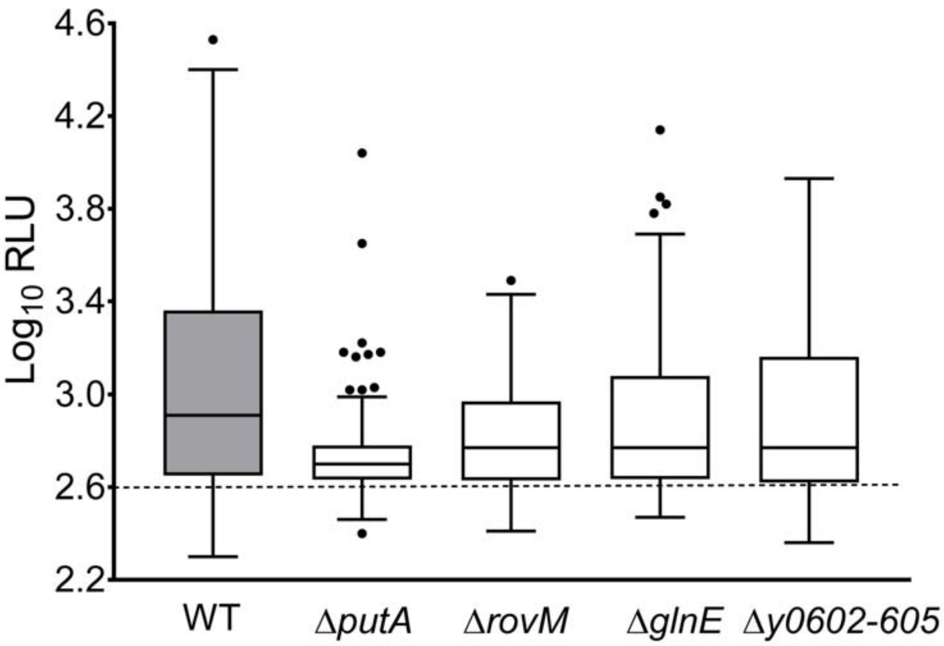

Figure S2. Bioluminescent *Y. pestis* mutants for which the range and the intensity of light emitted by infected fleas was affected. Box-and-whisker (Tukey) plots of the bioluminescence (in Log10 RLU) from fleas fed on blood contaminated with the bioluminescent *Y. pestis* strain of interest. The data correspond to the cumulative results of at least two independent experiments in which 96 fleas (48 males and 48 females) were collected after infection.

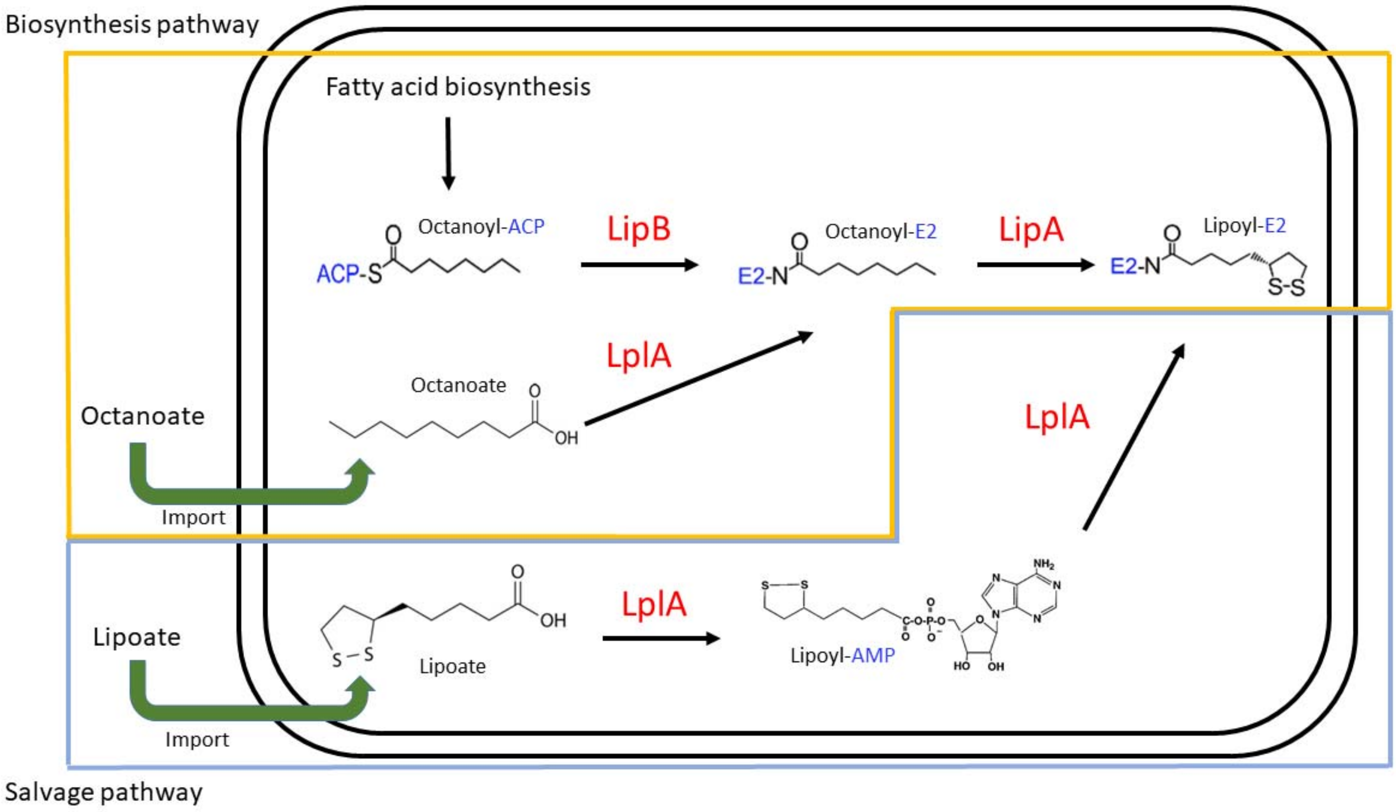

Figure S3. Lipoate salvage and biosynthesis in *E. coli* [11]. The salvage pathway involves the lipoate ligase LplA, which attaches lipoate scavenged from the environment to apoenzymes. The biosynthetic pathway comprises two steps. First, the octanoyl transferase LipB catalyzes the covalently attachment of an octanoyl group to acceptor proteins, using the octanoyl-acyl carrier protein from fatty acid metabolism. Subsequently, LipA inserts two sulfur atoms to from the lipoate cofactor. Along with its lipoate ligase activity, LplA is also able to ligate free octanoate to acceptor proteins. However, the physiological importance of this activity has not been determined.

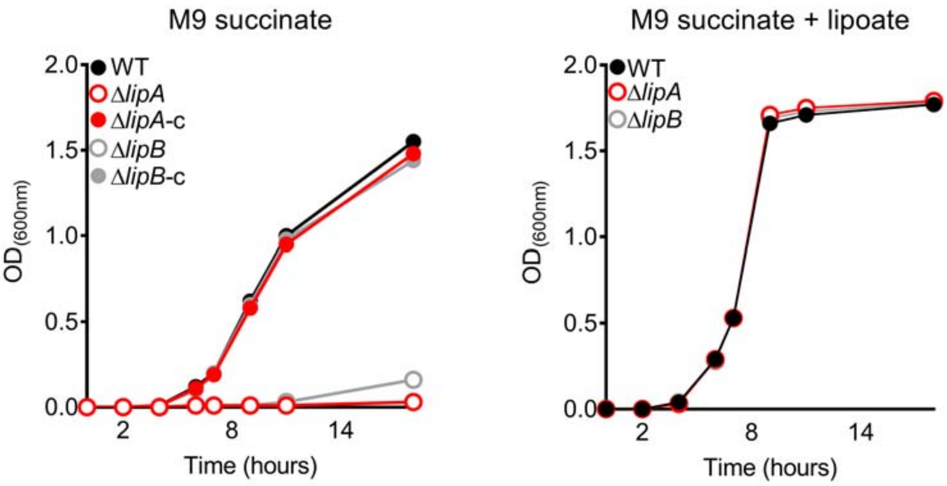

Figure S4. Growth of E. coli WT, Δ*lipA* and Δ*lipB* expressing (or not) *lipA* or *lipB* from *Y. pestis*. WT and mutant strains washed in the medium of interest (to reduce the carry-over of nutritional supplements) were cultured at 37°C with shaking in M9 + succinate supplemented (or not) with lipoate. "–c" indicates a mutant complemented with a *Y. pestis* WT copy of the deleted gene.

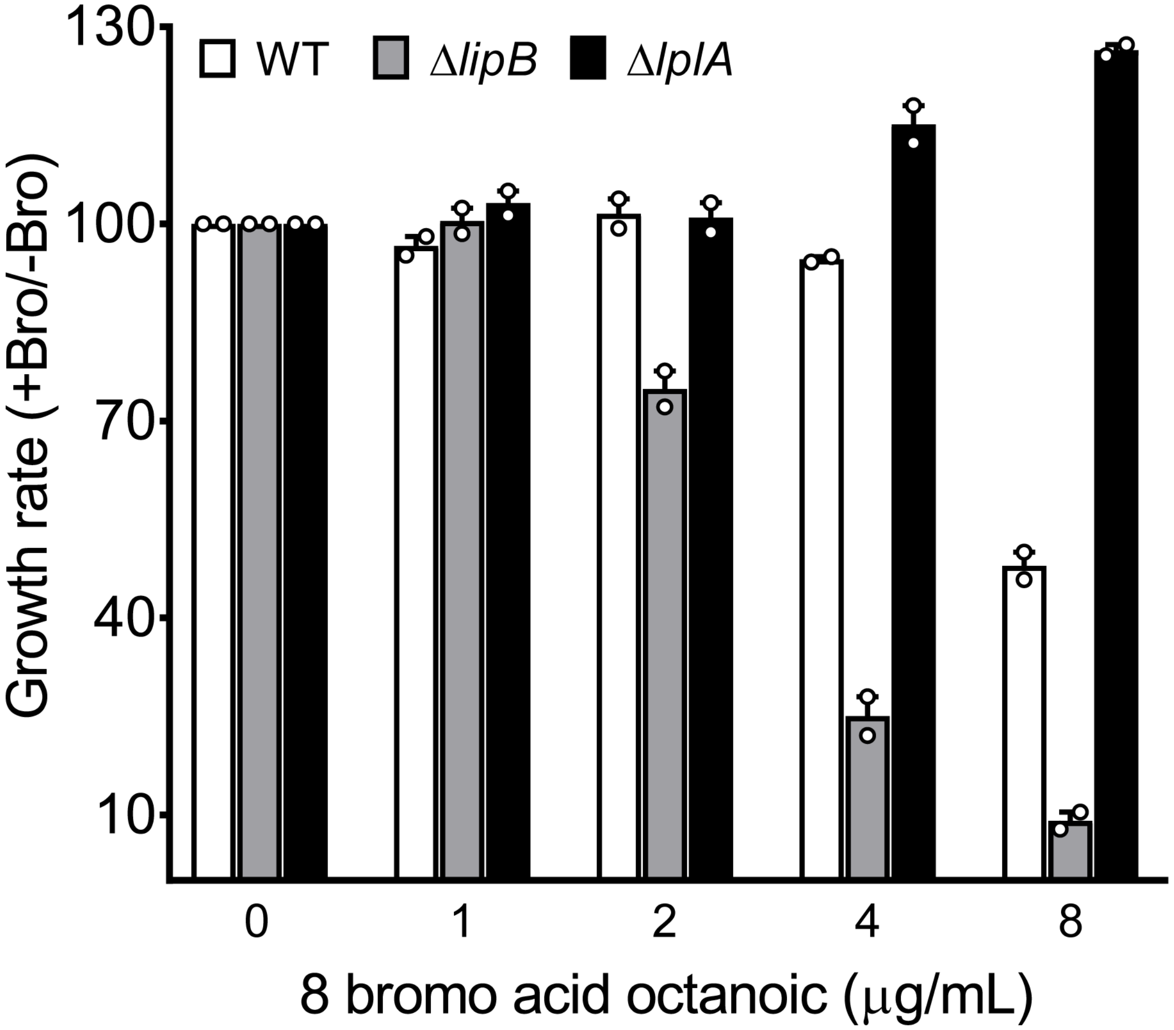

Figure S5. ***In vitro*** **growth of** ***Y. pestis*** **in blood in the presence of the lipoate analogue 8-bromooctanoate.** The bacterial growth rate (as measured by the ratio between the *Y. pestis* counts (for WT, Δ*lplA* or Δ*lipB*) in the presence [+Bro] vs. absence [-Bro] of 8-bromooacta-noate) was determined after incubation for 6 hours at 21°C in blood containing different concentrations of the lipoate analogue. The bars represent the mean ± SEM value for two independent experiments.

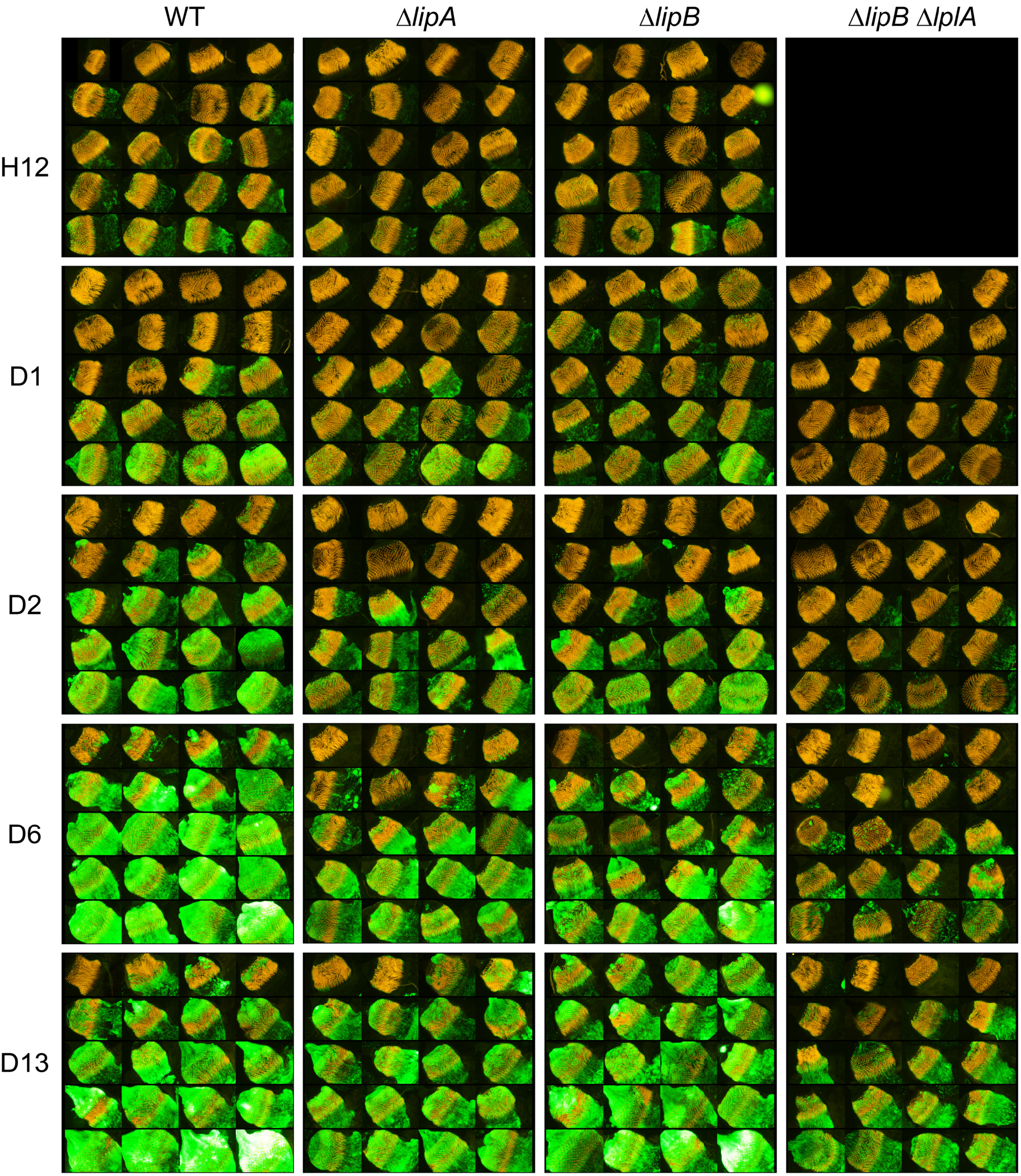

Figure S6. The time course of proventriculus colonization in fleas infected with WT, Δ*lipB*, Δ*lipA* or Δ*lipB* Δ*lplA* *Y. pestis* strains. Fluorescence images of the proventriculi (in yellow) infected with the WT, Δ*lipB*, Δ*lipA* or Δ*lipB* Δ*lplA* strain (in green) acquired 12 hours, 1, 2, 6 and 13 days post-infection.

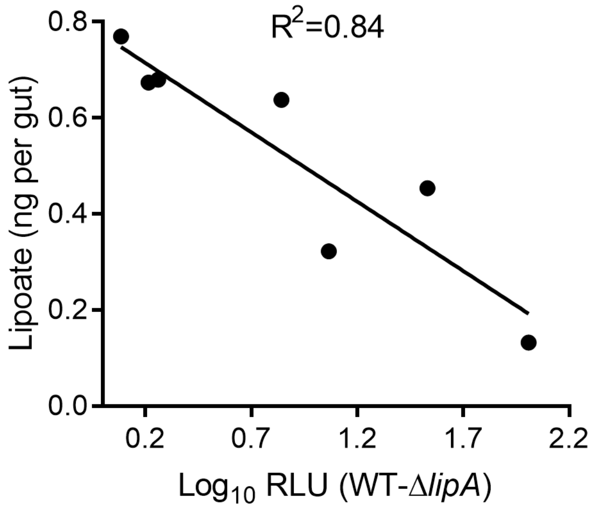

Figure S7. Correlation between the growth of *Y. pestis* requiring lipoate salvage and the amount of available lipoate in the flea gut. The amount of lipoate in flea gut content collected at various time points after feeding was plotted against the growth of *Y. pestis* requiring lipoate salvage in the gut. The data given in the figure are those from the Figure 6A. The trend line and the R2 value were calculated using GraphPad Prism software. The Log10 RLU (WT-Δ*lipA*) and lipoate concentration values change in opposite directions, i.e. there is an inverse correlation. Since a decrease in the Log10 RLU (WT-Δ*lipA*) corresponds to an increase in the *lipA* mutant's growth rate, the mutant's growth rate was positively correlated with the concentration of lipoate available in the flea gut.

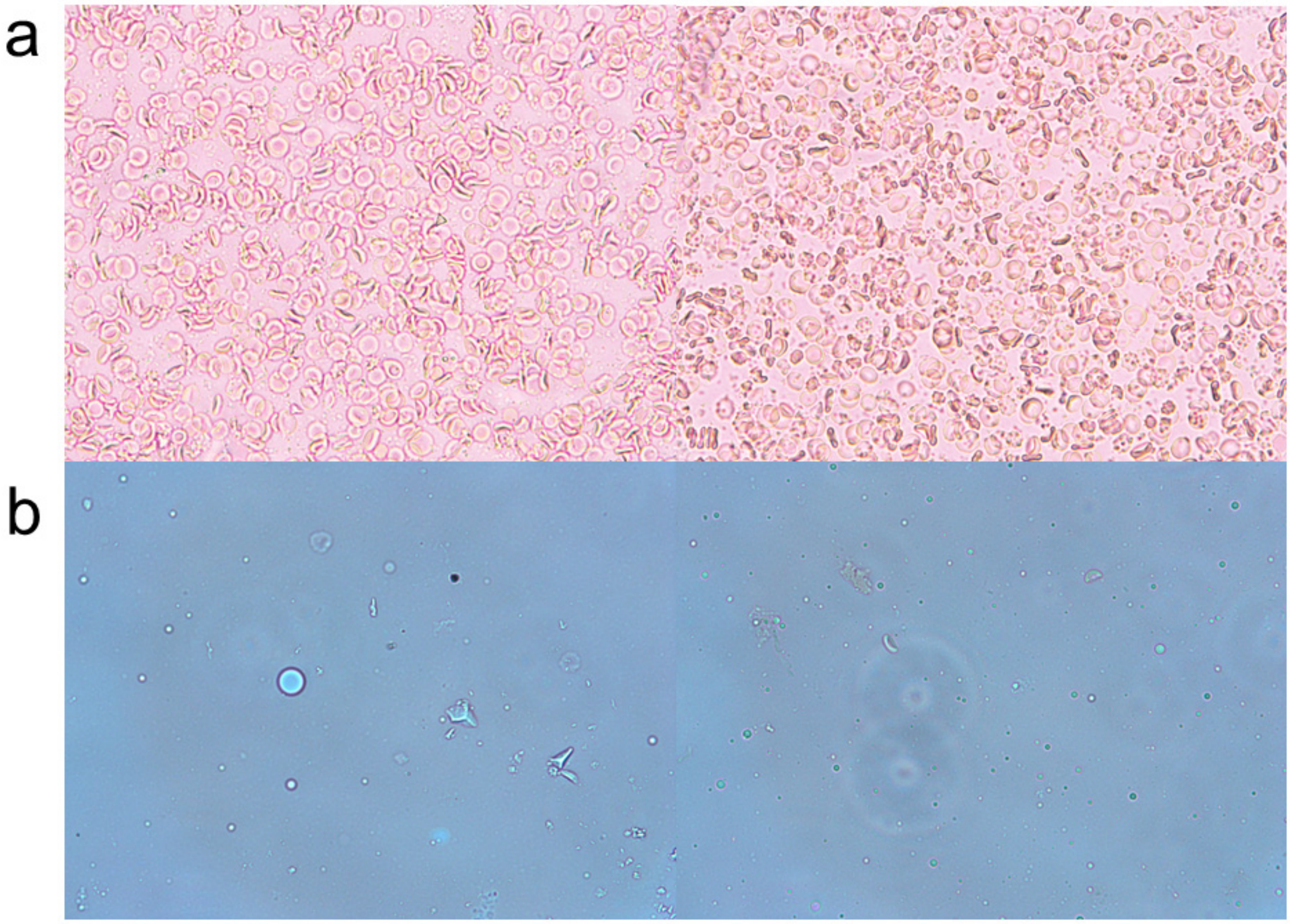

Figure S8. Flea gut content at different times after a blood meal. Flea gut content (A) immediately after feeding and (B) 6 hours after feeding. The photos are representative of the gut content collected from four different fleas.

Supplementary Text 1. References cited in Figures 2, S1 and S3 and in Table S1.

1. Wagner AF, Schultz S, Bomke J, Pils T, Lehmann WD, Knappe J. YfiD of *Escherichia coli* and Y06I of bacteriophage T4 as autonomous glycyl radical cofactors reconstituting the catalytic center of oxygen-fragmented pyruvate formate-lyase. Biochem Biophys Res Commun. 2001;285(2):456-62.

2. Zhang L, Alfano JR, Becker DF. Proline metabolism increases *katG* expression and oxidative stress resistance in *Escherichia coli*. J Bacteriol. 2015;197(3):431-40.

3. Taylor D. Bacterial tellurite resistance. Trends in Microbiology. 1999;7:111-15.

4. Arts IS, Gennaris A, Collet JF. Reducing systems protecting the bacterial cell envelope from oxidative damage. FEBS Lett. 2015;589(14):1559-68.

5. Perez-Gutierrez C, Llobet E, Llompart CM, Reinés M, Bengoechea A. Role of lipid A acylation in *Yersinia enterocolitica* virulence. Infect Immun. 2010;78(6):2768-81.

6. Dewitte A, Bouvenot T, Pierre F, Ricard I, Pradel E, Barois N, et al. A refined model of how *Yersinia pestis* produces a transmissible infection in its flea vector. PLoS Pathog. 2020;16(4):e1008440.

7. Erickson, DL, Lew CS, Kartchner B, Porter NT, McDaniel SW, Joner NM, et al. Lipopolysaccharide Biosynthesis Genes of *Yersinia pseudotuberculosis* Promote Resistance to Antimicrobial Chemokines. PLoS One. 2016;11(6):e0157092.

8. Nandineni MR, Laishram RS, Gowrishankar J. Osmosensitivity associated with insertions in argP (iciA) or glnE in glutamate synthase-deficient mutants of *Escherichia coli*. J Bacteriol. 2004;186(19):6391-9.

9. Vadyvaloo V, Hinz AK. A LysR-Type Transcriptional Regulator, RovM, Senses Nutritional Cues Suggesting that It Is Involved in Metabolic Adaptation of *Yersinia pestis* to the Flea Gut. PLoS One. 2015;10(9):e0137508.

10. Vadyvaloo V, Viall AK, Jarett CO, Hinz AK, Sturdevant DE, Hinnebusch BJ. Role of the PhoP-PhoQ gene regulatory system in adaptation of *Yersinia pestis* to environmental stress in the flea digestive tract. Microbiology. 2015;161(6):1198-210.

11. Yuan Y, Zallot R, Grove TL, Payan DJ, Martin-Verstraete I, Šepić S, et al. Discovery of novel bacterial queuine salvage enzymes and pathways in human pathogens. Proc Natl Acad Sci U S A. 2019;116(38):19126-19135.

12. Sikkema DJ, Brubaker RR. Resistance to pesticin, storage of iron, and invasion of HeLa cells by *Yersiniae*. Infect Immun. 1987;55(3):572-8.

13. Datsenko KA, Wanner BL. One-step inactivation of chromosomal genes in *Escherichia coli* K-12 using PCR products. Proc Natl Acad Sci U S A. 2000;97(12):6640-5.

14. Pradel E, Lemaître N, Merchez M, Ricard I, Reboul A, Dewitte A, et al. New insights into how *Yersinia pestis* adapts to its mammalian host during bubonic plague. PLoS Pathog. 2014;10(3):e1004029.

15. Choi KH, Gaynor JB, White KG, Lopez C, Bosio CM, Karkhoff-Schweizer RR, et al. A Tn7-based broad-range bacterial cloning and expression system. Nat Methods. 2005;2(6):443-8.

16. Sun Y, Connor MG, Pennington HM, Lawrenz MB. Development of bioluminescent bioreporters for in vitro and in vivo tracking of *Yersinia pestis*. PLoS One. 2012;7(10):e47123.

17. Hinnebusch BJ, Rudolph AE, Cherepanov P, Dixon JE, Schwan TG, Forsberg Å. Role of Yersinia murine toxin in survival of *Yersinia pestis* in the midgut of the flea vector. Science. 2002;296(5568):733-5.

18. Jones HA, Lillard JW, Perry RD. HmsT, a protein essential for expression of the haemin storage (Hms+) phenotype of *Yersinia pestis*. Microbiology. 1999;145(Pt 8):2117-28.

Supplementary Table 1. Strains and Plasmids used in the study.

Strains or plasmids	Relevant characteristics[a]	Reference or origin[b]
Strains		
Yersinia pestis		
KIM6+	pYV-negative strain, derived from KIM5	[12]
KIM6+ Δ*lipA*	derived from KIM6+, Tp^R	This work
KIM6+ Δ*lipB*	derived from KIM6+, Tp^R	This work
KIM6+ Δ*lipB* Δ*lipA*	derived from KIM6+, Tp^R Km^R	This work
KIM6+ Δ*lplA*	derived from KIM6+, Tp^R	This work
KIM6+ Δ*lipB* -Δ*lplA*	derived from KIM6+, Tp^R Km^R	This work
KIM6+ Δ*sucA* -Δ*sucB*	derived from KIM6+, Tp^R	This work
KIM6+ P_{glnB}	haboring miniTn*7::* P_{glnB} *luxCDABE* , derived from KIM6+	This work
KIM6+ P_{nlpD}	haboring miniTn*7::* P_{nlpD} *luxCDABE* , derived from KIM6+	This work
KIM6+ P_{pldB}	haboring miniTn*7::* P_{pldB} *luxCDABE* , derived from KIM6+	This work
KIM6+ P_{yjeH}	haboring miniTn*7::* P_{yjeH} *luxCDABE* , derived from KIM6+	This work
KIM6+ P_{ychJ}	haboring miniTn*7::* P_{ychJ} *luxCDABE* , derived from KIM6+	This work
KIM6+ P_{cysZK}	haboring miniTn*7::* P_{cysZK} *luxCDABE* , derived from KIM6+	This work
KIM6+ Δ*hmsHFRS* P_{cysZK}	derived from KIM6+ P_{cysZK}, Km^R	This work
KIM6+ Δ*ymt* P_{cysZK}	derived from KIM6+ P_{cysZK}, Km^R	This work
Escherichia coli		
DH5α	*supE , Δlac U169 (φ 80 lac ZΔM15) , hsdR , recA , endA , gyrA , thi , relA*	Invitrogen
DH5α (λpir)	λpir enables oriR6K vectors replication	Laboratory collection
BW25113	*lac* I^q *rrnB* $_{T14}$ Δ*lacZ* $_{WJ16}$ *hsdR514* Δ*araBAD* $_{AH33}$ Δ*rhaBAD* $_{LD78}$	[13]
BW25113 Δ*lipA*	derived from BW25113, Zeo^R	This work
BW25113 Δ*lipB*	derived from BW25113, Zeo^R	This work
Plasmids		
pCRII	cloning vector; Ap^R and Km^R	Invitrogen
pCR2.1	cloning vector; Ap^R and Km^R	Invitrogen
pCR blunt	cloning vector; Km^R and Zeo^R	Invitrogen
pKD4	ori6K vector, source of Km^R cassette, Km flanked by FRT sites; Km^R	[13]
pEP1013	vector encoding the Red recombinase, Ap^R	[14]
pEP1436	vector derived from pEP1013 and encoding the endonuclease I-*Sce* I, Ap^R	[6]
pEP1042	derived from pKD4, source of Zeo^R cassette flanked by FRT sites	[6]
pEP1087	derived from pKD4, source of Tp^R cassette flanked by FRT sites	[6]
pEP1237	derived from pKD4, source of Er^R cassette flanked by FRT sites	[6]
pEP1326	derived from pKD4, source of Sm^R cassette by FRT sites	[6]
pEP864	derived from pKD4, source of Er^R cassette (no FRT)	[6]
pEP865	derived from pKD4, source of Sm^R cassette (no FRT)	[6]
pEP866	derived from pKD4, source of Zeo^R cassette (no FRT)	[6]
pEP1446	derived from pKD4, source of Km^R cassette with I-*Sce* I site (no FRT)	[6]
pEP1454	derived from pKD4, source of Tp^R cassette (no FRT)	[6]
pUC18R6K-mini-Tn7(kanEW)	pUC18r6k-mini-Tn7 w/modified Kan cassette; Km^R Ap^R	[15]
pTNS2	oriR6K vector, helper for mini-Tn7 transposition, Ap^R	[15]
pLOU034	pUC18r6k-mini-Tn7(kanEW):: P_{tolC} *luxCDABE*	[16]
pLOU037	pUC18r6k-mini-Tn7(kanEW):: P_{cysZK} *luxCDABE*	[16]
pBT1	pCR zero blunt containing *lipB-lipA* under the control of its putative promoter, Km^R Zeo^R	This work
pBT2	pCR zero blunt containing *lplA* under the control of its putative promoter, Km^R Zeo^R	This work
pCH16	pACYC177 constitutively expressing *ymt;* Km^R Ap^R	[17]
pHms	pBR322 expressing *hmsHFRS;* Ap^R	[18]

a, Ap^R, Km^R, Tp^R, Zeo^R, Er^R, Sm^R resistance to ampicilin, kanamycin, trimethoprim, zeocin, erythromycin and streptomycin respectively

b, references are provided in text S1

Supplementary Table 2. Primer sets used in the study

ORF(s)	PRIMER SET (5' -> 3') USED TO		
	GENERATE THE MUTATION[a]	VERIFIY THE MUTATION	COMPLEMENTATION
y0046	ATGAGCCAAACCGCTAGTTCTACCTTAAAAGGCCAATGTATTGCGGAGTTGTGTAGGCTGGAGCTGCTTC	ATGTAGCCAATCCCGCCCTG	-
	TTATGCTTTACGTTCTGTGGTTATCGTTGTGGATTCTTCATCCTCAAGGGCATATGAATATCCTCCTTAG	GCTGCCTACATGCGACTCCA	-
y0063-y0065	TTAAGTGTGGACACTTTGTGGACGCGTTTCGGCAGCTAATCCACCCTTTAGTGTAGGCTGGAGCTGCTTC	GCCATGTCGAAATGGCCAAA	-
	TTATTGAGGCGACTTAGGGGCAGCATGGGTACGCATTAACGCCACACGGTCATATGAATATCCTCCTTAG	TAGAGCACGTCCGAGTAACA	-
y0090	GTGCCTGAATTACCAGAAGTTGAAACCAGCCGACGCGGGATCGAACCTTAGTGTAGGCTGGAGCTGCTTC	TTAATCCCATACCAGGCCAG	-
	TCAATGCTGGCAGTGGCGGCAAAAGAACGTGCTGCGCTGCCCGTGTTTCGCATATGAATATCCTCCTTAG	TGAACCGCCACTTAATGCCCA	-
y0095	ATGGCAGAAAAAGAAAATACGAAAAGGAACAGGCGCGAGGAAATATTGCAGTGTAGGCTGGAGCTGCTTC	GCGTATCGCTCAGATGGTAT	-
	TTATTGCAACTGGGCGACAATCAATGGCCAGCGGGAATCAAACTCTTGTGCATATGAATATCCTCCTTAG	GTCATTGCTATCGTGACGCT	-
y0177-y0179	ATGAGTTTGCTTCCGGTGATGGTGATTTTTGGCCTCTCTTTTCCACCGATGTGTAGGCTGGAGCTGCTTC	GCTAATATACTGTTGTCGGG	-
	TTAAATCAGCGCACTCTGATATTTACGCAACATCTCAGTCAACCGCTTAACATATGAATATCCTCCTTAG	GGCATAGTGTCAACCTCCTG	-
y0186-y0192	ATGCACGTCCAGGTACCCATCGATGATTTTTTTATAACCTGGGTGATCATGTGTAGGCTGGAGCTGCTTC	CCACTCGGGCGAGAAATAAA	-
	TTAGTGCCAACAAAGCATCGGTATATTAGTTAACCCTATTTTTGCCAATGCATATGAATATCCTCCTTAG	AATCACTCACGCGGTGAAAG	-
y0236	GTGTCAGAAGATATGTATCGCTATCGAGTTATGTCCTTATGCGTTACACAGTGTAGGCTGGAGCTGCTTC	ATGAGTGTCAGGGCCATATG	-
	TTAATACAGCGAAGGTTCGCCAATGGGTCGGGTTTTAAAGCGGCGGTGTACATATGAATATCCTCCTTAG	AATCGATCAGGGCTGGTTTG	-
y0260-y0263	TTGCATCGTATTACGATATCTAATAGCATCGCTATACGATGTGTATTCGTGTGTAGGCTGGAGCTGCTTC	AGAGTTCGTCTGCTGCGTTA	-
	CCTTTGAAGAATTTATCAATGGATTGGTCGAAGAAGACGATTTAGAGTAACATATGAATATCCTCCTTAG	CTTGCATGCGCTGTCTGAAA	-
y0329-y0332	ATGGAAATATTGTTAGAAGTCCGTGGCTTATCTGTTGAGTTTCCTGGTGTGTGTAGGCTGGAGCTGCTTC	TGCTCAAGAAAGACGGTACC	-
	GTGGATACCGAATTACTGAAAACCTTTTTAGAGGTCAGTAGAACTCGTCACATATGAATATCCTCCTTAG	ATTGAAAGCGTAGCCATGGC	-
y0350-y0352	ATGAAAAAAATTACTTTAGCTATCGCGTTATTCTCGGCATCTACAACGGTGTGTAGGCTGGAGCTGCTTC	CTACACGACTCGGTTACCTT	-
	TTAGCGGCGGCAACTCGCGGTTATATGAATGATATCTTGCTCGGTATCCTCATATGAATATCCTCCTTAG	CGCATTGGTGCTGAGATCAA	-
y0404-y0406	ATGACGAACAGTTCTCCTTATCTAGAAACGGATGTCATCATTATCGGTGGGTGTAGGCTGGAGCTGCTTC	GGGGGATGGCGAAATTGTTA	-
	TCAAGCCAATGCCCGCGCCAGTAAAGTAATCGGGTGCTCACATTTCTTACCATATGAATATCCTCCTTAG	GACATTGCAGATTACGCTCC	-
y0421	ATGAACCTCGGCGCTATATCCACTTTATTACTATGGCTTCTCGCGATGAAGTGTAGGCTGGAGCTGCTTC	GAAACAAGCGGTGTAAACAG	-
	TTACCGCCGCCAGCCTTGTGCTTCATGGCTAAATCCACACGCGAGCATAACATATGAATATCCTCCTTAG	TCCGGCACGATAAAGAAGGG	-
y0434-y0439	ATGTTTAATAATTCCATTCATAAAGTGTCGATCTGTATCGCGCTAACACTGTGTAGGCTGGAGCTGCTTC	AGTATTAATTGTTAGCGGTC	-
	TCAGTGGCGTGGTGACGGCGGCCAGTACCGTTGAATCACCAGGCTGGCGGCATATGAATATCCTCCTTAG	GAGTGCGAATTATTCAG	-
y0440-y0441	ATGATCGAACTGAAACACTTACGCACCCTGCAAGCTTTGCGTAATACTGGGTGTAGGCTGGAGCTGCTTC	CTGGCACTAAATCAACACCCGC	-
	ACGCTTCAGTGACTGGTTGGCGCTGTTCTCCATCACGGACGGCAGCGTATCATATGAATATCCTCCTTAG	CTGCTTCCGCTACAATTGCATT	-
y0508-y0510	ATGACGATGAGCCAAATACATAAACATCCTATTCCAGCTGCAATTGCAGAGTGTAGGCTGGAGCTGCTTC	TATCAACCTCCAAAATGCGG	-
	TCAGTGAGCGCCGCCTTTAGATGCCCCCAAGCCGGTTTGTGAACGGACAACATATGAATATCCTCCTTAG	CTATTCCCTGCGCAAGCAGT	-
y0511	ATGAAAAGTATAAAAATAAGTCTGGCATGGCAAATACTCATTGCACTGGTGTGTAGGCTGGAGCTGCTTC	CCTGGCGAAGAGACTATTTG	-
	TTAGCTTTGATTAGCTGGCGTCTGCTCTACCGCAAACAACGTTTTTTCATCATATGAATATCCTCCTTAG	GTCGAAACACATCGGCTGAA	-
y0534-y0535	ATGAGCTGGCCCGAGTTTAAATCTCAATATCTGGTGCGCTTTTGGGCACCGTGTAGGCTGGAGCTGCTTC	GATGATCCACCGCGCTAGCT	-
	TCAGCGCTGAATCAAATAGCGAATAGTTGGCCCATCCTGCTGAATATCCACATATGAATATCCTCCTTAG	TTTCCGCGCCAGACAAAGC	-
y0539-y0547	ATGAATATTGATCTGATGCCGTATTATGCCCAACCAGGGCCACTGCCATTGTGTAGGCTGGAGCTGCTTC	TCACTCTGATGGGTGGGGCT	-
	TTATTGCCGCAGATAAATCTGTGGCAGGGCGCTTTCTGGGTGACGCGAAACATATGAATATCCTCCTTAG	TGCTTCAGTTTTGCGGGTGA	-
y0555-y0560	TTGTTCAATAATTTCATTCTAAAAGGAAACGTGATGATATCGTTAAGCAAGTGTAGGCTGGAGCTGCTTC	GATAATCCCCCCATGAACCA	-
	TTATACCGACACACCATGCTGGCTTGCCAGCGCAGACAGACCACCGGCAACATATGAATATCCTCCTTAG	AAACAAACTGGCTGCCTCAG	-
y0575	ATGTTAGAAATTTTCTTTGTGATCGGTTTTTTTATGGTGCTTATGGTCACGTGTAGGCTGGAGCTGCTTC	ATTTTTACGGCCGCATTGGC	-
	TCAGTAACGCCGTATCACCGGTTTTTGCATTGACCGCCAAATCCAAGCCACATATGAATATCCTCCTTAG	TGCATATGTAGGGGTACAGC	-
y0602-y0605	ATGATAGCCGTAGAATTAGTCATCGTTCTGCTGGCCATTTTTCTTGGGGCGTGTAGGCTGGAGCTGCTTC	AAAATCTGTGCGGAAACCGG	-
	TTATTTTGTAACCGCGGGGGTGTTTTGTAAATGCTGTAAGAATTGCGCAGCATATGAATATCCTCCTTAG	GATCAAACTGCGGCAGAGCA	-
y0700	ATGGACGATTCTATGATTGATAACACCGGTTCTGTAGTCGATAAAAATACGTGTAGGCTGGAGCTGCTTC	TGCTGCCAGAGTAATAGCCT	-
	TTACCGGCGCGGGGTATGCATCAGATCTTGCTGTAAGCTGGCAACGGTTTCATATGAATATCCTCCTTAG	TAAAAACCAGCCTTCGGGCT	-

y0705-y0706	ATGGAGCTGCTGTGTCCTGCGGGTAACTTACCTGCATTAAAGGCCGCAATGTGTAGGCTGGAGCTGCTTC	AGTTTTCCCAACACAGGCGG	-
	TTACGACACCAGTTCTAACCCGGCAACACGATGCCAGTAGCCGTTACAATCATATGAATATCCTCCTTAG	GGGGTATCTGACGTCGCCAA	-
y0732-y0733	GTGAAAACAGTAGTGATTAAACGGGACGGCTGCCAGGTACCTTTTGATGAGTGTAGGCTGGAGCTGCTTC	TGATGTGCTGCGGGTTGCCT	-
	TTAACGTAAACAGTGAATAACCTGATTGCTGCTGCCGCGCCAGATAAGTGCATATGAATATCCTCCTTAG	GGGTTTGGGCTAACGTCGTG	-
y0773	ATGAACCGCCTCCCAACCCTGTATGGCATTATTGCGATTTTACTGTGGAGGTGTAGGCTGGAGCTGCTTC	GTCCCTAACACATCAGGTCA	-
	TCAAGTACGAAGGGGATTTCGGCTGGCTGACCAGCATAGCAACGAACCTGCATATGAATATCCTCCTTAG	TGAAGAAGTGATGGTGCGTG	-
y0857-y0859	ATGACTCATATTTCTGATGTTGATAGAGACGTGCCGCTTATCACGTTAAAGTGTAGGCTGGAGCTGCTTC	AATCAGCGAGGGAGATAAGC	-
	TCACTTGGCGTGACATTTTTTTAGATAATTCAAACGGTTCTGCCTCATGGCATATGAATATCCTCCTTAG	AGCTTTTCGACCCAACTGGT	-
y0864-0867	ATGAAAGAGACCAACGAATCCCGGCTGTTAACGACAGGTGTCACCCGCCGGTGTAGGCTGGAGCTGCTTC	ACTCCCCCCTCCTCGAAAGA	-
	TTAGCGATATAACTCAACCCCAGCCGGTGTCACTTGCAATTCATGTTGCCCATATGAATATCCTCCTTAG	CGCCTTGGCGTTTCTGAAGA	-
y0915	ATGCGGCTGCATATCTATCTTCGCCGATTGCTTATCGCCCCATTGCTCTTGTGTAGGCTGGAGCTGCTTC	ATGGCGCTTGGATTTGGAGA	-
	TCAATTCGCTGCCCGATAAAGACTATAACCAGTAATAGCGCCAGCGATATCATATGAATATCCTCCTTAG	GTATTGATAACCCCATGCCC	-
y0932-y0935	ATGAACATAAAAAAGCGGATGTTGTGGCTGTGGGCAGTACTGGTATTGGTGTGTAGGCTGGAGCTGCTTC	CACCTTTCAGAGCACGTCAT	-
	TCAGGCAGGGCCAGATAAACCGCGGACTGACGATATAAACTGTTTCAATTCATATGAATATCCTCCTTAG	CGATCAACATTGTCCAGCGA	-
y0948-y0949	ATGTGCGAATTGCTTGGGATGAGCGCAAATGTGCCCACGGATATTTGCTTGTGTAGGCTGGAGCTGCTTC	CGGTTATGCCGATCGTATTC	-
	GTGGCAGTATCCAAAGAGCTAAAACAGCAACTACTGGGGTCATCGGTTTACATATGAATATCCTCCTTAG	CTGACTGATAATCTGCGCCT	-
y0982	ATGGAATTACCAGTTAAAAATAAGCTTAAACGTGGCCTGACGACACGTCAGTGTAGGCTGGAGCTGCTTC	TCTTGATGCCGTGAAAGCGT	-
	TTAATCAGCATTCTTGATCAGCGTAGCCTGTTTTTTCTGATACTTGACCTCATATGAATATCCTCCTTAG	GAGTAACGAACATAGCCAGC	-
y1036	ATGTACCGTTTAGCCGCTTTTGATATGGATGGAACGTTGCTGATGCGTGAGTGTAGGCTGGAGCTGCTTC	TAGTTGCTGAATCAGGGTGC	-
	TCAGAATTCGGGGGAATAGGTGAGGTGTGGTGAACTTAGCCAATGGTGCACATATGAATATCCTCCTTAG	GGTCAGCAATAACATTGGGG	-
y1043	ATGAACTCAACCATCAGGGGCGACAACATGAGTCAGGCATTAGAAAAACTGTGTAGGCTGGAGCTGCTTC	TAATGCCTTTGGCTGCCAAG	-
	TCAACGATGTAAACGAATCACACCCTCCTGCACAGTAGAAGCTACCAGCACATATGAATATCCTCCTTAG	GTTAGTTGTTAGCCTGTCGG	-
y1067-y1068	ATGTCATTAAATGTTAAGCTGCATCCATCAGGTATTATTTTTACTTCCGAGTGTAGGCTGGAGCTGCTTC	AGGCCCTGTTATGGTTGTAG	-
	TTATTCCCATACCTTCCAAGGTGCCCTGCCTTCTTCCCATAGTTCATGCACATATGAATATCCTCCTTAG	TCTGGTTGGAATTCGCGGAT	-
y1076-y1077	GTGATATTAAGAAAGTTTTTTATTATTTGTGATACAGTCTATCCCGATGAGTGTAGGCTGGAGCTGCTTC	TTGCTTGGAGAAAAGGTGGG	-
	TTATCTTATGGCAACTGATTTAATTTTCACCCTAGCTATCCCAAATAAAACATATGAATATCCTCCTTAG	GACAGTTACCCCTCCCATAT	-
y1113-y1114	TTGCGAAAATATGATCGAACAGGTGCAGAAAAAACTTCGCTCATTGCGTAGTGTAGGCTGGAGCTGCTTC	GCTGGTAGCATTGAATGCAG	-
	TTATTTCGATGAACGTAAAATCAATGTGTTGTCTCTTACTGCAACGACTCCATATGAATATCCTCCTTAG	TAACATGCCGTCGATGGCAT	-
y1115-y1120	GTGACATTGTATGACATTAAACCTCAGTTTCAGAATCTGCTGCGACCGCTGTGTAGGCTGGAGCTGCTTC	GACACCGACCTGTTGACGCA	-
	TTATAGCTCCAGGATCTCTTGAATCCAATCATCCCAGCACTCGCTGCCAACATATGAATATCCTCCTTAG	TGGCGCCAATCCGTATTGAT	-
y1140	ATGTTTTTCAGAGCATGGGATAATTACCCGAACACATCAGATGGAGGGGTGTGTAGGCTGGAGCTGCTTC	TCCATCGCAGCCGAAATTAG	-
	TCAGCCTGGATTAACTGACTCTGCGGGTGATTTTTTAGGTTTTAGATAACCATATGAATATCCTCCTTAG	CGCGCTGATAGTAGGCTAAT	-
y1147	ATGACCATGGCACAGGCGCTGGTCAGGTTTCTTAATCAGCAATATGTGGCGTGTAGGCTGGAGCTGCTTC	GAATATGGTAATGGCACGGC	-
	TTAGTACTGACGGGCACGTTTTACCTTCATCTGGGTTCGCTCTGCAGCCTCATATGAATATCCTCCTTAG	GATATCTCGGTGGTCTGGTA	-
y1150-y1155	ATGTCGCAACCTTTATTGAAAATCACCGATATGGCGAAAAGCTTCTCTGGGTGTAGGCTGGAGCTGCTTC	ATGGTGACAGGTTTCGATGC	-
	TTATGTAATAGGATAATTATGATTTATCCAAACATGATCGTTTTCCCAGCCATATGAATATCCTCCTTAG	CAGCGACAACAGGCCAATAT	-
y1160	ATGTCAACATTAGAGAAGCTGGTTTCTGCATATTGCCATACCAGTTTGGAGTGTAGGCTGGAGCTGCTTC	TCATACTCGCCGCTGTGGTG	-
	CTACAACGGCCTGTAAATATCCCTGTAGTACGTTAATCTTTTACGAAAAACATATGAATATCCTCCTTAG	AGTACCGTTCGTGAAGCCCG	-
y1161	GTTGTTTAAAACATAAAGGTGTCGCCATGAAAACCAGCCATCGGGCAATCGTGTAGGCTGGAGCTGCTTC	GTGGTTAATCATACTCGCCG	-
	CTACACTGATATTAAATAAGCCACCTGCGTGATGTGAACCTCTATCGTGGCATATGAATATCCTCCTTAG	CGTATTTGCCGCCTCAGTTT	-
y1169-y1170	ATGCGCCAAAATGCTAAACAGACTCGTGAACTCATCATCGTTCACTTACGGTGTAGGCTGGAGCTGCTTC	AGCCTTGTTAGGGCGGTTAT	-
	TCAGTCTTTGCGCTCAACCGAAGCTGCGGCTTGGGAGTCAGATGCCGCTTCATATGAATATCCTCCTTAG	AAGCAGAAGCCATGGCTATG	-
y1251-y1252	GTGTCTTTTGAACATCAAAAGGTGTTGGGTATGGAAAAGAAAAAAATTTAGTGTAGGCTGGAGCTGCTTC	GCCATTTTGCGATAACCATC	-
	TTAGAATTTCAGGCTGTTAGCAATATCGTCTTCGCTTTCGACAGTACGGTCATATGAATATCCTCCTTAG	TGGCCCGCGTTGATGATAAG	-
y1265-y1267	TCAGTAAAAGTCACGACCTGCCTTTTCTGATTGGGTCAGCCAAACCGGTTGTGTAGGCTGGAGCTGCTTC	TAGTTGCCAAGAATCTGCGG	-
	TCAATGTGCTAACCAGACTTGTAGATGTTCAGCGACCGGCCCCAGGGCCACATATGAATATCCTCCTTAG	GCCCACTTAATATGTCCAGC	-
y1282	TTGGTTTTACCAAATAACCAAGATAATTATCTTCCCTTGGAATTTATAGCGTGTAGGCTGGAGCTGCTTC	ACACTCTCCTTGGTTTCTGC	-
	TTACAAACTTTCAGTGAAAGTCCGGGTAATCACATCGCGTTGCTGTTCTGCATATGAATATCCTCCTTAG	GGCGTCTTCTTTCTTTGCCT	-

y1288	TTGGTGACTAATGTGGGCGAACAATTAAAAAAACAACCGGTATTACGTGGGTGTAGGCTGGAGCTGCTTC	GGTTACAGTCATGGTCTTCC	-
	TTAATAGTTTAGATAGAAATCACTGATGAGCTGGCAAAACTCAGGTGAGTCATATGAATATCCTCCTTAG	CCATAAGATTGAGGGCGTCT	-
y1359-y1361	GTGGATGCATTATGTCCGGTTTGCCAGAAGGCGATGACTGAGGTGAGTGGGTGTAGGCTGGAGCTGCTTC	TGGCTTACTGATGGGGTTTC	-
	ATGTCGCAATCTTCCGCCTCCTCAATTTTTACTGTCAGCCGCCTGAATCACATATGAATATCCTCCTTAG	TGCCAAGTTCTGCCGTGTTA	-
y1390-y1393	ATGGCATACCAGCAGGAGCGAATCATGAGTCACCGTGAATTGGAACAACGGTGTAGGCTGGAGCTGCTTC	ATAGATAAACGCAGCGCACG	-
	TTATGGGGACACTGGATGATGGCGTAAACGCTCACTACGCCGCCGGAGCACATATGAATATCCTCCTTAG	CCTCTCAGTGAGTTATCTCG	-
y1442-y1447	ATGACTGATTTATCGCGGCGTAAATTGCTGACGGGTTTTTGGCAAACCGGGTGTAGGCTGGAGCTGCTTC	GATTTAACTGGGCAAAACCC	-
	CTAAAAACCAATAGGTACATCTTTCATATCCGGCAACTTATGGGCAATCCCATATGAATATCCTCCTTAG	GTACCGAGGTTCCCTCATCT	-
y1453-y1454	TTGAACACCAATAGTAAAAAAACCTACCTTAACATTGCTATTTCATTCATGTGTAGGCTGGAGCTGCTTC	AATCCGCATGCGTTCCCATA	-
	ATGAACTTAAAAAGTAAAATCTTTATTTTCTGGGGGATGATGGATCTCTACATATGAATATCCTCCTTAG	TTTGCCACACTCTTGCCACA	-
y1474	TTGGCCGTTTTACAAATTTTTGACAACTTTATACAGCAAACAGACAACTTGTGTAGGCTGGAGCTGCTTC	CAACCTCACCATCCTTTTCC	-
	TTACTTCTGCTGCTGTTTGACGTTGTAGTTCTGTTCTTTCAGCTCTTTACCATATGAATATCCTCCTTAG	GAAACAGGCTTTTTGACGGC	-
y1524-y1526	ATGAAAATACCTATACCCTCTATTGGCCATGTTGCTTTACTGGCGTCTGCGTGTAGGCTGGAGCTGCTTC	GGAAAATGCCGTTAAAGCCG	-
	CTACTCGGCAAAAATATGTGCCTGGCCCATGATATCCAGGCCAACAGTTACATATGAATATCCTCCTTAG	TCCAAGAACTGAGGCTCCGC	-
y1554-y1556	ATGTCAAGGACAATAAATTTGAGCAGCCCGGCTATGCCTTATCTATTGGGGTGTAGGCTGGAGCTGCTTC	TATTTACCCGCTTCGCGACT	-
	ATGCTCTACGTGTGTTTCCCACGCAGAACATGCTTACCTTTTATCTATAACATATGAATATCCTCCTTAG	CGTCTTATCACTACAACGCG	-
y1561-y1562	ATGGAAAAGAAAAAACAGTTTTTACCGGCGTTGTTAGAGCGTTTACTCGAGTGTAGGCTGGAGCTGCTTC	AATGGCATCAACCGACTGAC	-
	TCAGCGCGCTCCGGACATAACATCGATAATAACGATTATAGAAAGTAGGCCATATGAATATCCTCCTTAG	ATCTGCTGGCTGATGGTGAT	-
y1567-y1576	ATGCTGGAAGCAAAAAATCTGACCTGTATCCGCGATGATCGCTGTTTATTGTGTAGGCTGGAGCTGCTTC	TCGGTTTTCCTGAACTGGCC	-
	TCAATCGGCGGCATCTATGCTATCCAGATCGCCCTGAATAGCCTGAGCATCATATGAATATCCTCCTTAG	CGGCCCCATCTAACACCACA	-
y1595	ATGAATGTTTTATTTCCATTGTTTGCGGTGATTATTTGGTCAGTCAATGCGTGTAGGCTGGAGCTGCTTC	GGATCCTTGAGGTGACTACA	-
	CTATCTACTGTTTTTGTTGTTTGGGATATGTCCAGCAAGATCTAAATTGCCATATGAATATCCTCCTTAG	AGGAAATAAGGAAACCCGG	-
y1608-y1610	ATGCTGGATGGATATTCCAAACTGATATTTGAAGGTGCGCTGGTGACACTGTGTAGGCTGGAGCTGCTTC	ATTCTTCGGTGTAGGGACTG	-
	TTATTTCAGTGAGCCAGAAAGAAACTGCTGTAAACGTGGGCTTTTAGGGTCATATGAATATCCTCCTTAG	GGGTGAATCCGCAATATTGG	-
y1626	TTGAACAGCGAACTACTGTGGGTGTTGACGCTACTGCTGATAGCTATTGTGTGTAGGCTGGAGCTGCTTC	GCCACTTCTCATGGTGCGGT	-
	TTAGCATTTATTTCTGGTAATATTTTCTCTCAGCACGTTTATAGGATCCACATATGAATATCCTCCTTAG	CGCCAAAGCCCAGAGATGGA	-
y1629	ATGACAAATGCAAATCGTCCGATAATTAATCTCGACCTCGATCTGCTTAGGTGTAGGCTGGAGCTGCTTC	CCATCAAACGGTAGGGCTAA	-
	TTAATCTTCATCACCTGTCAAATAATCGGAATCCAATATTAATGAAGATTCATATGAATATCCTCCTTAG	GATCCAGGACACGTTTTGAG	-
y1651	ATGAAACACAGCAAACAAGCCGTATTGATAATCGCTCCGGTAATGGACTAGTGTAGGCTGGAGCTGCTTC	AACTGGTCACGTTACCGGTA	-
	TTAATAGGTAATTGCTGTAGGTGCCGTTTCGCCAGAGAAATGGGCCAAAACATATGAATATCCTCCTTAG	TGACGCGCTATTACTCATGG	-
y1659	GTGGTCGTGAAACAACTTTCTGGTTTGCTGGGTGAATTACGACAGAAATTGTGTAGGCTGGAGCTGCTTC	AATGCCGCTTGCCAGATGAT	-
	TTAGGCTTTAACGGGCTGCGCTTCGTGCTCCAACAAGCTTTGTAAACCTGCATATGAATATCCTCCTTAG	GCCCAACGACGGTTAAAAAC	-
y1660-y1664	ATGTCGACAAGTGTTTTTAACCGTCGTTGGGCGGCACTGCTACTGGAGGCGTGTAGGCTGGAGCTGCTTC	AATGCCGCAATTAACCGCAC	-
	CTATCTTTCGCCAATCTTCTCCCCTTCATACTTGCCGCGCAGCCAGTCGGCATATGAATATCCTCCTTAG	GCCTGGTAAAAGCCCAGAAT	-
y1666-y1667	ATGAAAAACATCAAAAATTTCGTTGCAGTTATCGCATTGTCTACTCTTTCGTGTAGGCTGGAGCTGCTTC	TTGACACCACCAGCCCCTTT	-
	ATGGGCTTTTGTATTTCCAAGGTGTTGGCAGCTATTTATTGCATCACTGACATATGAATATCCTCCTTAG	ACCACCAGAAAAAGCCCATC	-
y1673-y1675	ATGAAGTCACTTGTCAAAGTTTCATTGGCCGCACTGGCGCTGGCCTTTGCGTGTAGGCTGGAGCTGCTTC	GCAAGCTAAGTGATTGGGGT	-
	TCAGGAAACGTGTTGCAGGAATTCCCGTAAACGTTCGCTGGGCGGATTGGCATATGAATATCCTCCTTAG	ACGAGCGGTGGCTATTTCTA	-
y1696-y1699	TTGCGTTATTGTTATTTTATATTACACACGGCTATATTATTTCCACTGTTGTGTAGGCTGGAGCTGCTTC	GTAATCCGAAAGGGAGAATG	-
	ATGGACGTATTCGGTACTGCAACAGGTGGCGGAACCGGTATTGGCACCGCCATATGAATATCCTCCTTAG	CGCTGATGTGATCCGATCAT	-
y1700	ATGTGGGTGGTGCTGGATCGGGTAACTCCTGGCGGTCATGGGCCGGAAGAGTGTAGGCTGGAGCTGCTTC	AGTAAGATCCCGACACCGAT	-
	CTAAGCCTGATTGCCCTTCAGAGGCTCGAATGTTCCTCCTGGCCTACTGGCATATGAATATCCTCCTTAG	GCCCAACCGTATAAGATAGC	-
y1737	TTGCACAATACCCTATCTAACAAGGCGGGTGGCAGGGATAGATTGAGCTGGTGTAGGCTGGAGCTGCTTC	TTGGCGCATTCCTCCTTGTT	-
	TTACTTGCAACGATAATCGTGATCAGTAAAACCAGAGGTAATGAGGCCGGCATATGAATATCCTCCTTAG	TGATTGCCCAAAAGAACGGG	-
y1783-y1784	ATGGGCTGCACAGTTAAGCGAGTGGCATTAGCATTAACGCGGGTTTCTCAGTGTAGGCTGGAGCTGCTTC	TACCACAACCCAACAGCGTT	-
	ATGTCCCAACAATATCGTTATTCATTGCCTGAACGACGTGGTGATACCCGCATATGAATATCCTCCTTAG	CGATAAATAGGGCGACAGGT	-
y1791	ATGGACAAATTACTCGACCGATTTTTCAACTATGTTTCTTTTGATACACAGTGTAGGCTGGAGCTGCTTC	GTCAATCAACACGTCCCGGC	-
	TTATGATTCTTTAGCCCGCTTGGCGGTCAGCTCAGAAATTCGCATAATGACATATGAATATCCTCCTTAG	CTCAAACGCCCCTTTTCGG	-

y1806-y1807	TCATACTGACCGCTGCTGCCGTTGTTCCATAATCCGATGCAAGTTTGTCTGTGTAGGCTGGAGCTGCTTC	ATTGGCGCGCTGCTGAAAAT	-
	TTAACGTGTCGCCAGTGTTGCTGTTATTACCTTGGCATAAGGGGTGGTTGCATATGAATATCCTCCTTAG	TCTGCACCTGTCTACCATGA	-
y1820	ATGGAGAGAATCATGTCATTAACTCCTTTTGTTATGAATTTATTACTGGCGTGTAGGCTGGAGCTGCTTC	CAGGCACTAACACCGGTTAT	-
	TCAGGCCGGTAATTCAGATGCAATGCGCCAACGTATGGCACTGACACTACCATATGAATATCCTCCTTAG	CTTGGGGATTTACTCGGTTG	-
y1823-y1827	GTGTTAGTTATTTTGGGTTATCTCGTGGTCTTAGGTGCGGTTTTCGGTGGGTGTAGGCTGGAGCTGCTTC	TCCGCAGTCTGCCGATATAA	-
	CTATGCGGTAACCACCTCATCAGCCGCGACGCGTTTTTCACGGTTTAAGGCATATGAATATCCTCCTTAG	GCCTCGGATCTCTTGTACTT	-
y1835-y1836	ATGGAGTATTGGCTGTTAATTTTTTTACTTATCTTCTTGTTTGTCATCTAGTGTAGGCTGGAGCTGCTTC	AACTTTTCTACACTGCCGCC	-
	CTAATTCAATGAGACCACACCATAGAAAGCGGCCACGGCAGCAGCACTCACATATGAATATCCTCCTTAG	AGCAGGCTGGTAACCACTTT	-
y1846-y1847	ATGAAAAGTATCCAAAAGTGTGACTGTAATTATCTGGTCAGATTTGCAGTGTGTAGGCTGGAGCTGCTTC	TGGTTGTACTGCTGCCTCTT	-
	TTACCATTGATAACCGACGCCGACACCAACCCCCATATCGCCTTGTGTATCATATGAATATCCTCCTTAG	ACCCTGTGCGGCTCTATTTT	-
y1849	ATGCACGTTCGTTTTCGTCATTTATTCTTATTACCATTAATAACTTTGGTGTGTAGGCTGGAGCTGCTTC	AACTTAACGCCATGTAGCGC	-
	TTAATCTACCAGAATACGCTTATTGGTTGCGCCCCACAGAATTGACATTTCATATGAATATCCTCCTTAG	GGTGTAAGCCTCTTAACCTC	-
y1860-y1862	ATGAGGAAAACCTTATTTACTTTAAGTGTAATAGCCATATTACAGACAGCGTGTAGGCTGGAGCTGCTTC	ATTGCGTCTTGTTGGCGT	-
	TCAGTAAGTGACAGTAACCAATAACGTATCTAGATAAACTCCCGCAGTAGCATATGAATATCCTCCTTAG	ATGGCAGTCTGAGCGTTGTA	-
y1880	ATGAAATTAAAATTAATTACTCTGGCAGTGGCAGCAGCAGTATCCGTGTGTAGGCTGGAGCTGCTTC	CTGCCATTTATTTGGTGGGG	-
	TTAGAAACTGTATTGGATACCTACGCGGTAACGAGTTTGGCGTTCGTCAGCATATGAATATCCTCCTTAG	CGGTGGCTGCAATATCACAT	-
y1898	GTGAATTCAAAAGTCGGTTGCCTAATGGCAATTTTATTGTTGGCGGGGTGGTGTAGGCTGGAGCTGCTTC	TTGTAAACAAGGCTGCAGCC	-
	TTACTCTGACATCGCCCGGTAAGCGGTATCCACCTTCCACAAGTAACGTGCATATGAATATCCTCCTTAG	ACTCAGCTCAGGAGCGATTA	-
y1901	GTGGTGATGAGTCACTCAGGTCAAGCCATTGATAAAAAATCATGTCTACCGTGTAGGCTGGAGCTGCTTC	TGCGATAGATCATGCCTCTG	-
	TCATTTAGCCAGAAAATAAAACGCCCCGATTGAATAAACAATCAGGGCGACATATGAATATCCTCCTTAG	AGCTTGGGTGTTGGATATGC	-
y1959	ATGAACAAAATACAGCATACACATGTAGACACCCGAGAACATCTACTCGCGTGTAGGCTGGAGCTGCTTC	CAAAAATGCCGGAGTCTGCA	-
	TTAGGCTGGTTGTAATATGAGTTCAATGCTTCCCAGTGCACAAGTCAGTGCATATGAATATCCTCCTTAG	TGGCCCGTTGGCGATAATAT	-
y1980	ATGGGTATTTTTTCTCGCTTTGCCGACATCGTGAACGCGAACATCAATACGTGTAGGCTGGAGCTGCTTC	CGAGCAGATTATCCAGTTGC	-
	CTATTCAACTGATTTCATTTTCGCTTTTAATGCCGCTAATTGGCTACTGACATATGAATATCCTCCTTAG	AACGCCATTTTGCTGACGAC	-
y2044	ATGAAAAAAATCACTTTGGCACTGCTCGCCGTGGCCTCGTTAGTACCTACGTGTAGGCTGGAGCTGCTTC	TTCTAATCGACCGGTTGCCC	-
	TTAAAAACGATAACCTGCGCCAAACATAAATACCCATGGGGCCAAACGGGCATATGAATATCCTCCTTAG	CGCTCGGTACCGGGTGTAAA	-
y2068	ATGATGAGTGAGATATCTACCTTAACCATTAAAATTCCTCTGGAATTAAAGTGTAGGCTGGAGCTGCTTC	AAGACAATAAGCCGTTGCCA	-
	TTATTTTTTCTTTTGAGCGGTTTTCGCACTGTCTTTCAGCAGTTGACGCACATATGAATATCCTCCTTAG	GGGACATGGTTGGTGAGCAA	-
y2097-y2098	GTGCAACCCAAACTTTATAAGGAAGCCATCATGTCAGCACCCCATTCTGCGTGTAGGCTGGAGCTGCTTC	ATCAACGGAGTTGATGCCGT	-
	TTATATCGTCCGTTTAGCTAACTGTTTGTAACGGTCGAAGATAACCGCCGCATATGAATATCCTCCTTAG	TGGCAGCAAAGGATTGGGTT	-
y2111	ATGTTAACGCGTTTATTTGTGACAGGTACCGACACTGCTGTTGGTAAAACGTGTAGGCTGGAGCTGCTTC	GCGCTAACGCAAGCTGTCTG	-
	TTATCCTGAAATAGCTGTAAGATCTAAATATTTTGCTAATGGTTTTTCTTCATATGAATATCCTCCTTAG	GGTTTGTGAAATGACCGGCG	-
y2112	TTGATGTTGTCTTGCATGAAACGGCCAATCCATCTCTATTACTACCGTGCGTGTAGGCTGGAGCTGCTTC	GATACCGATACAGGCCGTAT	-
	TTAGTGAGACCGATATACCGATATTGGGCGCAACCAGCGGGCTATTGTTGCATATGAATATCCTCCTTAG	TAATCCTGGCTTGGCCCATT	-
y2121-y2123	GTGGCAAAATCATTTTTACGCAGTGGAAGTTTGGACGATATTCTGGCGTTGTGTAGGCTGGAGCTGCTTC	TCAATGGCCTCAACAACTGC	-
	TTAATCATTTGCGGGTTTTATCACCGATAATATTGCCGCCAGTTGTTGCCCATATGAATATCCTCCTTAG	ATGCGTCTTCGCTGAAAGTG	-
y2129	GTGGCGAAGAGAAAATCTACCGGTACCGGCTGGGTTATCCTACTGATAGTGTGTAGGCTGGAGCTGCTTC	ATGTATTGAGCGCCGCTACT	-
	TCAGGCCAATAATGCAGCCGAGACATCACGATTAATTGAAATAACCGCATCATATGAATATCCTCCTTAG	ATAATCGACCCACAGTGGCT	-
y2180-y2227	TCACTGTGATATGGCGCGGGGCATATACGGGGCATCACCCGTAAATCTTGGTGTAGGCTGGAGCTGCTTC	TTGCATCAAGTTAACGCGGG	-
	CTAGGCCTGGGTGTGCGTACAGGTATACTGGCTAGCCTGCACCTCATCAACATATGAATATCCTCCTTAG	TGCCCTGAGTGTGCCTTGGT	-
y2272	ATGGCTTTGGTATCGCAAGCTCGAAGCTTGGGTAAGTATTTTTTATTGTTGTGTAGGCTGGAGCTGCTTC	CTGTTATAGGGACATCGCGT	-
	TTAGCTGCTGCTCAACATGGCAGATTCAATCCGCCGTCGATTGAATTGCCCATATGAATATCCTCCTTAG	GGATCTACTTAAGCCACCCT	-
y2275	TTGGCACTGCTGATGAGAAGAATTCCTGCTATCATTAGCGGACTATATTTGTGTAGGCTGGAGCTGCTTC	CGCTTACGCGTATACACACT	-
	TTACAGCTCTGCTAGCTCGGGGATCGCTGGGCGACCACGCTGCTCCAGATCATATGAATATCCTCCTTAG	AGGTGAAACACTGCACTCTG	-
y2289-y2290	ATGAAGTCTTCTATTGTTGCCAAACTGGAAGCGTTACAAGAGCGCCATGAGTGTAGGCTGGAGCTGCTTC	AACGATCCGTGACTATCGCT	-
	TTACACGGCCCATTGCCCCAATGTCACCCGGTCATTATTGCCATAATCTTCATATGAATATCCTCCTTAG	ATGTTCCCTGCGGACTGAAT	-
y2296	ATGAAACGTAAGAGCGCAACAGTACTGGGTAATATGCTGATGGGGTTGGGGTGTAGGCTGGAGCTGCTTC	ACTGGAATACAGGAGAAGGG	-
	CTATGAGTGACGTTGATTCTTGGTACAACGCTTATCAAAGTGCTTAACCCCATATGAATATCCTCCTTAG	CCGTTGTCCGCTGAAATCAA	-

y2313-y2316	ATGTCGAAAGTTACTAGTCCTGATTCTATCAATAGAATAAAACATACGGAGTGTAGGCTGGAGCTGCTTC	GTTCTCGCTCAAACTCCGAT	-
	TTAGCGGGGTTTTGGACTCATGCATGGCATGCTTCCAATACTGTGAGAAACATATGAATATCCTCCTTAG	CCGGCTTGAGTCACTAAGTT	-
y2320-y2321	ATGCAAGGACGCGATTACATTCCCTGCGATATCGCAGAGTACCATCTGCTGTGTAGGCTGGAGCTGCTTC	AATAGATGACGTCGCCTTGC	-
	ATGACTGCGCTGAACATAGCATTTTGTGAACATCATTTCAATTTTAATGACATATGAATATCCTCCTTAG	CACAACCGGTCACCACAAAA	-
y2331-y2335	ATGTCGACATTATTAATTTCAGCACCTCATCTGGCGTTCAAAATTATCGCGTGTAGGCTGGAGCTGCTTC	ACGTCAGGATAGTGGCGGTA	-
	TTACGGTTGGCAAAGTGCAGGCGTCGGATACGAAAATAGTGCCAGAGCGTCATATGAATATCCTCCTTAG	TAACCCAACGCTGAACTTCC	-
y2340 – y2341	ATGATTCGGGTGACAAATCTGCCGAGTGCAGCTAACACCGCAGCAACGTCGTGTAGGCTGGAGCTGCTTC	TTGCTTGTTGATCCGCCGTA	-
	TCAACGGTAAGTTTGCTCACCCCACGTCAGCATGGTTGTGATGAACTGCTCATATGAATATCCTCCTTAG	TTCTGTGTCGGAAACAGGTG	-
y2350	TTGAATTTTATCTATAACAGATGGCTTATGACACGATATGAACAGCTAGCGTGTAGGCTGGAGCTGCTTC	CGCCGTTACCTTACCGTTTT	-
	CTATCGACGTTCGTTCGTCAATTGGCCGATTAACTTCGCCAAAATACGAACATATGAATATCCTCCTTAG	GAGAATGCCTCAATCAAGTG	-
y2362-y2370	ATGCGCATTTGGCAAAAATTCTTGCTGCTCTTCTGCTGTTGGGTGTTATCGTGTAGGCTGGAGCTGCTTC	TCTATCTTGAAGGGCGACGGG	-
	TTAGGGAGTTTTAGGTTTCGCGGTTTTCAGATGCTGAATGTGGTCAGCCACATATGAATATCCTCCTTAG	CAGAGGTGTTTTCCATCGTGA	-
y2412	ATGCAGAGCGTGGAGCTCAAACCAGATTTATGCAACCCGCAACTATTGGTGTGTAGGCTGGAGCTGCTTC	CGTGATGGGCTGGTCAATGA	-
	TCATTTTTTATCTTTACAATCTGCTAGCCTCAACTGCACGCTGTTACGTCCATATGAATATCCTCCTTAG	AAGCCGTTTTCAACCGCG	-
y2437	ATGGTGCAATACCGTTTGAAAAAATCTCGTTTATATATGAGTCAATATCCGTGTAGGCTGGAGCTGCTTC	CACGCCATAACAAGAGAGCA	-
	TTACTTGAATGCATGGGCGCGAAAATTGATTGGTTTAATAAACTCTGTCACATATGAATATCCTCCTTAG	CTATGCTGTTTTTCGCGCTC	-
y2455	ACAACGTATTGACCGCACACCACACTGGCTCATTAAACAGGCTATTTTCAGTGTAGGCTGGAGCTGCTTC	AACCCTGAATTCGCCTTACCAA	-
	TTGCACTTCAGTAGGCAATTGAGGCAACAGTGCTTTCTGTACGTCATTCTCATATGAATATCCTCCTTAG	GGGGGCTGATAAGTGAATCGGT	-
y2463	ATGTCAGGACTCCAGTTAAAAAAACGGCCGCTGAGCCGTTATTTAAAAGAGTGTAGGCTGGAGCTGCTTC	AGCGATAAAACGCTTACGCG	-
	TCAATAGTTCTTTTGCCATGCCAAATACTGATCATATTTACGCAAAGCAACATATGAATATCCTCCTTAG	CCCCCCAAGGAATTGATGAA	-
y2466-y2468	ATGGCAAGTATCAGTGCATTAGGTACCGGTTCAGGACTGGATTTAAATACGTGTAGGCTGGAGCTGCTTC	ATGATTTGGGCTTACTGCCC	-
	TTATTGTGTTTCACCCAGCAGCAGTGCCTGATCGGGGAACTGACCATAAGCATATGAATATCCTCCTTAG	CTTTGAATAGCAGGCTGGCA	-
y2538-y2546	ATGAAGATTGTTAACCATTGGATTGATGGGAAAAATATTACCAGCAATGAGTGTAGGCTGGAGCTGCTTC	TGGTTATTCGCACTTGGGTC	-
	TTACACCATCACCGGGTGAAAATGGCGTTTAAAGTAAATCAGACCATGGCCATATGAATATCCTCCTTAG	TGAAACAACCCAAAGGGGAG	-
y2556	ATGAATCAGTCTCTCTCCATAAGTACGGAGCCGAAACGTTCGGCTCCTCGGTGTAGGCTGGAGCTGCTTC	TGTCCTCCCTGCTTATCGCC	-
	TCAGAAGTCCATGGAGACGGATAATTTCAGCGTACGTGGATCGCCCTGATCATATGAATATCCTCCTTAG	CGCCGGGGGATTTCAGTAAA	-
y2566	ATGATTAAATGGTATGAAGAAAGTGATAGTGAAGTGAACCGAAGCATTGCGTGTAGGCTGGAGCTGCTTC	ACTACCAACAGGCAGTGTCA	-
	CTACACTTCCTGGCTGAGTAGATAACAGATCATCGCCGCGCGTAGCGGGCCATATGAATATCCTCCTTAG	GGTGACAACTTCAACGGCAA	-
y2571	GTGGAGACGAGTTTGTTTGATGATAATAAAGTCATCGCGTTGATTGGTTTGTGTAGGCTGGAGCTGCTTC	CATTCCAGGCTGTCGTTGAT	-
	TCAGTCCTCTTTATAACAGTTCTGCACATCAATAAGTTGTGCATCATTACCATATGAATATCCTCCTTAG	CAGCTAAGGAGCAGTGATAG	-
y2586-y2587	ATGGCTCGTTATTATCTCTCTGCGATGGATGTTTTATACCGCCTATCAATGTGTAGGCTGGAGCTGCTTC	CGGTGTGCAGTATCATGAGA	-
	TTAACCGTTATATATTCCAAACATCCGTGGCAACCACAGAGAGATTTCCGCATATGAATATCCTCCTTAG	TATTGGGGGAGAGAAACAGC	-
y2591-y2592	ATGGCTAAAGAAATATTCTGTGCGTTTGGCGTTGATGTTGACGCGGTGGCGTGTAGGCTGGAGCTGCTTC	AACTAGCGGGTTGTATCACC	-
	TTACTTCACACTCTGCGGTGCCACGTGGTGGGCAAACAGCATAATGACGCCATATGAATATCCTCCTTAG	CGCTAAGGATGCCAGTTACA	-
y2598-y2600	ATGAATCTGAAAGGGAAGAAAGCCTTGGTCACTGCTGCCGGGCAGGGGATGTGTAGGCTGGAGCTGCTTC	AGGGTATCACGCGCTTTGAA	-
	TTAAACGCGAAAACGGTACTTCTCTATCGACGATTGATGCATTTCGATGGCATATGAATATCCTCCTTAG	TTCAAGCTGCTGGTGTGTTG	-
y2601-y2602	TTGACCCAGATTTCATTTTTTAATGCCCTTATTAGCCTGACCTTTAGTCCGTGTAGGCTGGAGCTGCTTC	TTAACGTAGCCAACGCACAG	-
	GTGACCGTAATAAAATACAGTGACTACAATTTTCGAACATCGTTAAACGCCATATGAATATCCTCCTTAG	TGATCAGCGCAGCCGTTAAA	-
y2632-y2642	GTGTCAAATACAGCACAGTTAATTACACCTTCCCCTATAGGGCTAATTGCGTGTAGGCTGGAGCTGCTTC	GCACAACAAGTCATTAGC	-
	ATGGTTTCACAACGTCAATTCAATGAAAATAATATCCAACATCTTAATGGCATATGAATATCCTCCTTAG	GGTAATGGCCTTTACTAT	-
y2660-y2661	ATGGCCGATATTAATACAGCACAACCTCGTGACTGGTTACTGGAAATGAGGTGTAGGCTGGAGCTGCTTC	ATTATTGGCGTGCAGGAG	-
	CTATTTCTTCTTCGCATACTTCAGTGAATCCAACGCAACCGCAAAGATGACATATGAATATCCTCCTTAG	CCCCCAGGGAAGCTATTTTT	-
y2770-y2771	TTACTGATTCTCTTCCTCCTTATTGCCTCCTTTCTTGGTATCTTGTGGATGTGTAGGCTGGAGCTGCTTC	TGGGCACCCAGTCAGCTTGA	-
	CTAATCCAACCAAGGGAGAAGCTGCCGAGCTGCATTATCAAATAAAGGGCCATATGAATATCCTCCTTAG	ACAGCCAACGGCAGGACAA	-
y2785-y2786	ATGTTTACAAAAAGTAATTTCAAAAAATCGGTTGTTATCATCACGGCAATGTGTAGGCTGGAGCTGCTTC	CTTTTTTGCGAAGGCTACGG	-
	TCATATGCCGATAGATAAACCCGTTAATAAACTAACACCACGTTGAGTATCATATGAATATCCTCCTTAG	ATAATTCGGGTTGCCGCAAG	-
y2787	ATGAAATATATAAAGCTAACGGTACTCGCCGGAATATTTGTAGGGATCAGGTGTAGGCTGGAGCTGCTTC	CTGATGCCAGAGCCGCTTTT	-
	TCAATATGTGTGGAATAGTTTTTGAATTTCTTGTGGTTTTTGAGTTTGCGCATATGAATATCCTCCTTAG	GGGGCGTCCATTCCATTACAA	-

y2826-y2833	ATGAAAAAGAAAAATATTGCCCTCATCCTCCCTCTCGCTCTTTTACTTAGGTGTAGGCTGGAGCTGCTTC	GTTAACACACTATGCGGTGG	-
	TTATACCCCAAATAATTCGAGTGGCAGGAAGGCAGCCAACGCATAGGCAGCATATGAATATCCTCCTTAG	TCGGCATTCTCATCGCCATT	-
y2857	GTGAATATAAACGTCGCAAATTTGTTAAACGGCAACTATATCTTGCTGTTGTGTAGGCTGGAGCTGCTTC	CTTACTACTGGCTGTCTTGC	-
	CTAACCTAATATCCCTGGCCAGAGGATGACAATCAACGAGCCAGCCAAGGCATATGAATATCCTCCTTAG	ACCTGCCATACTGCAAACTG	-
y2862-y2863	GTGCACGCCATTTACCGCAATATCAATGAAAAAAAGAAGAAAAATAACAGGTGTAGGCTGGAGCTGCTTC	CCGTAGAAATCGATAGACCG	-
	GTGTTCACAATGGAGATTTTCATGGACACTACGCAAACAAGCACTCTTGCCATATGAATATCCTCCTTAG	CGCCATCAAAAACCAAGCTG	-
y2878	ATGGCGACTTCTCAGACAAAAGAACTCCCCCGCCCTCAGCTGTTTACTGTGTGTAGGCTGGAGCTGCTTC	ATGCTCCCTCGCCTATCGCT	-
	TTACACAAGTGCAATATGTTGCACAAATAAGTTGATACCGGTGCCACCGACATATGAATATCCTCCTTAG	CGGATACGGCCCTTAAACGG	-
y2880-y2882	ATGGAGACTGTTCTCATGAAAATGAAATGTTTTGCGAAAAATGCGCTGGCGTGTAGGCTGGAGCTGCTTC	TTAGTGGTGATCGGGAGTTG	-
	TTAATGGCATACTCCGCTGAGCATATACAGCCCGGTATCACCTTTTTCCGCATATGAATATCCTCCTTAG	ATTTAACAGCCGGGTTGACC	-
y2889	ATGGATAAGGCAACCGAGACCCGCCGCGAGGGAGCACCTACCGCGAGCAAGTGTAGGCTGGAGCTGCTTC	TCTGCTGATGGTTGATGAGC	-
	CTATCCGAAGTAACGGATCCGGAAAGCATCAATGGCGACAGCGATAAAAACATATGAATATCCTCCTTAG	CGAAAAGTGTCAGCAATGGC	-
y2892-y2894	ATGAGTGAGTATCATGAAAAAAATAATGCACTCTTCAAGTCGATTACCGCGTGTAGGCTGGAGCTGCTTC	AGGGGTCTATGTTTAGACCC	-
	TTATCGCAGTTGAACGTAGGAGGAACCGTCAAAATAGCCCACCACCATACCATATGAATATCCTCCTTAG	TCGAGTAAGGGATCGAAAGG	-
y2895-y2897	ATGCTGCCCACTTCACTCACCAAGGCCAAACTTGTTGGCCGAGTCTGGCGGTGTAGGCTGGAGCTGCTTC	ACCCCTGCTGGTTCGAACGA	-
	TCAGATGCTGGTATATCCCGCATCGGCAGTAATGATTGAACCTGTCAGCACATATGAATATCCTCCTTAG	CCGTGAAGAGGCGTTGGTGT	-
y2909	TTGTCATTACTGTATCGCAGCAGTGAAAATAAATTAACAACACAAAGTGGGTGTAGGCTGGAGCTGCTTC	AAAGCGGTAGCGCTATGCAA	-
	TTAAGCCAGTAAGTTTTTGTTACTCATCTTTTCATGACAGAATTTAGCTACATATGAATATCCTCCTTAG	CCAATCGCCGACAGAGTAAT	-
y2928-y2929	ATGAGCTTCAACCTGCCCGGAATGTATTTGTATTACCTTTCAAGAGATAAGTGTAGGCTGGAGCTGCTTC	AAGCAGCCAGCCAGATGAAT	-
	CTACTGGACATTAGCTTCACGGATCTCTGCCAATATAGCGGCAAGAAAATCATATGAATATCCTCCTTAG	TACTATCGGTTAACGTGCGC	-
y2949-y2950	ATGATGATGATCCCTGAACAGCGTCGTGACTTTATCTACCGCTACGTGCAGTGTAGGCTGGAGCTGCTTC	TACTATGTGATGCGCATCGG	-
	CTATGAAAACGCCTTGATATTTTTATTTTTTCCCAACCATCCCAACCCTGCATATGAATATCCTCCTTAG	CGCTGGGTGAGTCTCATTAA	-
y2958	ATGTTTTCCTTTCTATTAGGACACTCTATGCTTGATTCCATTCGCTCACGGTGTAGGCTGGAGCTGCTTC	CACAGTACATCTGCACACAG	-
	TTAGATCTTAAATTGATTAACGGTATCAGTTAAATCAGCGGCCTGAGCTTCATATGAATATCCTCCTTAG	AATTACACCAACCGGTGACG	-
y2961-y2963	GTGGCGGTAAACATGAGTAAACAGATTTCAACAAGGCATACGTTGGCTTAGTGTAGGCTGGAGCTGCTTC	CATTATGGTGGTAAGAGGGC	-
	ATGAATAATTTACATTACAAGGACGTATTGACTGGAAGAATGCCAAATGCCATATGAATATCCTCCTTAG	CATTCCTACAGCTCCACTGT	-
y3035-y3037	GTGGAGAACAACATGAAGAATCAATACGGTAAAGTGAGTTACTGGGTAGCGTGTAGGCTGGAGCTGCTTC	CCGAAAGAACGGAATACCCT	-
	TCAATCTGGTACAGGGCCATTCTGGCGGTTAAATGACACACTTTTTATCTCATATGAATATCCTCCTTAG	TGAATCTCAGGGCCTGAGTA	-
y3043-3046	ATGTACGTTCTGGTAACAGGTGGTAGCGGTTACATTGGTAGCCATACCTGGTGTAGGCTGGAGCTGCTTC	CTTGCAACAACGCCTCGGTC	-
	TTATAAAGGCAACGCAATAAAGTGATAAACCGTCGTTGAGCGATAGGTCTCATATGAATATCCTCCTTAG	GCGTAGTCGGCAGCAGTGAA	-
y3154	ATGTCAAAACGATTACCACCCCTGAATGCCTTACGGGCTTTTGATGCCGCGTGTAGGCTGGAGCTGCTTC	TGTCTGGCATACCAGACCAT	-
	TCAGTTTTCATAACGAAAACGTAATTTCTCCTGCTCGCTGGCAGCTCTTGCATATGAATATCCTCCTTAG	CCCCAGAGAGGTGCTTAATA	-
y3183	TTGCGGGTAATCTTATTTTCCGCAAAACTGAGTGATGTAAGCGGTTACCCGTGTAGGCTGGAGCTGCTTC	AATTGCAGGCATTCTCGCAC	-
	TTAATCAGTGTCATCCCGTAGGCTTGGCGGGCTGGCCACAGAGTGGCGGCCATATGAATATCCTCCTTAG	AAGTTGGGGGTAACGGATGA	-
y3222-y3223	ATGATTGGGATTGTTGTTTCTGGCCACATTAATTTTGCTTCTGGTATGGCGTGTAGGCTGGAGCTGCTTC	AACAGTGGAGCTACAAACCG	-
	TTAGGCTGAGTGGCGATAAGCCCATTCACCTTTAATCAGGGTTGCGGCAACATATGAATATCCTCCTTAG	CCCGTATTACATCCGGTGAT	-
y3226-y3228	ATGGCACAGCCACATACTCAACAAGCCTATTTTCACATGATGGCCAAGCCGTGTAGGCTGGAGCTGCTTC	TGTTCTGTTTGCCCGTTCGC	-
	TTAAAAGAGTGACGTCATTTTTGAGCTGTAGCTGGTTACGCCTTTGCGGACATATGAATATCCTCCTTAG	AGCCTCCGATCTGTGCCTGA	-
y3249-y3250	ATGTTTGGTCATTCAAAACACGATCATCGTCATGGTGGTCATCGCCATGAGTGTAGGCTGGAGCTGCTTC	ACTGATATTGTCACTCGGCC	-
	CTATTCTGCATGATCACTACGGTCACGCGGTGAACCTTTCTTCAGTAGGACATATGAATATCCTCCTTAG	CCTGAATGATCCGGTCTCTT	-
y3289	GTGGAGAGTGGCATGGAAAACATCATAAAATATGATATTAATTATTTTTTGTGTAGGCTGGAGCTGCTTC	CTTGAAGTATGGCGGGTGTA	-
	TCACATAAAGCTGAGAAGTTGCGGTGCCAGGGATAAATAAATCATCACTACATATGAATATCCTCCTTAG	GTATTACCCGTGATGTACGC	-
y3302	ATGACTCAGGATGAACTTAAAAAAGCGGTGGGCTGGGCAGCATTAGATTAGTGTAGGCTGGAGCTGCTTC	GGCAATGCTGAGGTATTCAC	-
	TTAGCCAATCACTTTAACGCCATTGGCCGTACCAATCAGGGCCACATCAGCATATGAATATCCTCCTTAG	TCGAATGAATCCGGTCAGCA	-
y3389-y3390	ATGGCTTTGCTGGTTGGGATGACGATCATTATTGCTGCTGTGGGTATTATGTGTAGGCTGGAGCTGCTTC	CCTCCCTGTAACTCGACTTA	-
	TCACACCGATAAATCCTCAGAAAAAACCAAAGACAACGCCTCTACCGGCACATATGAATATCCTCCTTAG	GTTCAGTGCACGCTTGTGAC	-
y3418-y3423	TTGTCTCAGCGAGGTTATTGGGTGATCACCGCTGGGAGGCGGGAACATCCGTGTAGGCTGGAGCTGCTTC	CAAACTACTTTGCCGGGGAT	-
	CTATTCACCCAGTCTCCCTGGTTGCCAGGCTTGTGCTTGCTGTTGTAATGCATATGAATATCCTCCTTAG	CAACGTTGAGCTATGGGTAG	-

y3435	ATGGCTGACGCACCTTTATCGACTGCTGCACCGCCCTCAATCTCCAGGCGGTGTAGGCTGGAGCTGCTTC	AATTGGGCACGGCTCCTAAT	-
	TTAATACCAGAGATACCAGGCTAACAGCGAGACAACGATGATGCAGATGACATATGAATATCCTCCTTAG	ACTGAGTGATGATGCCGTGA	-
y3474-y3475	TCAGCTCTTGCAAATCGCCAGTTTATCGCCGGAGAGGGTATTGATGTTCAGTGTAGGCTGGAGCTGCTTC	GGCGAATAAGCGTGCTTCTA	-
	TCAGGAGACTTGTTCGGTTGTCAGCATCTCACCACTCACCAAAACGCTATCATATGAATATCCTCCTTAG	TAATTTCCCGGTCGATCCGA	-
y3509	ATGAGTAACGTACTAATTATTAATGCAATGAAAGAGTTTGCCCATTCTAAGTGTAGGCTGGAGCTGCTTC	ATCGGCACCGCAAGTTCATT	-
	TTAGCTGTTAACGTTTTCGGCTAAGTGCTGACGATAGCGGGCAATATCCCCATATGAATATCCTCCTTAG	AAGGGATTGCCGAACTCTCT	-
y3519	ATGAACACCTTTCGTATGCCTGCACTGTTTCTCGGCCACGGTAGCCCGATGTGTAGGCTGGAGCTGCTTC	GTCAACACCGGGTTGTTGTT	-
	TTAGCCGATTTGCACCGACAACATACTTAACGACCCCATCTCAACCCCATCATATGAATATCCTCCTTAG	GACGGGTATACCCAAAGTCA	-
y3525	ATGCGAAGGGGATACAAGGAATCTGATATGTTGCCACTCCCTTCGGAATTGTGTAGGCTGGAGCTGCTTC	CCGTATATCGATGCCTGGTT	-
	TTAGCTCACATTAGCGTCGTTTGCCGCCAACCATTTATGCCAACTGGCCGCATATGAATATCCTCCTTAG	TCGACTTTCACTACAGGCAC	-
y3569	GTGATGACAGGAGTGATGTTACATAACGTCAATAAAATATATCCAAACGGGTGTAGGCTGGAGCTGCTTC	AAACAACGTGAGTCAACCGC	-
	TTAGATATAGAGAATATTTTTTTCGCTTTCTTTATCGAAAAGATGGCACTCATATGAATATCCTCCTTAG	ACTGGCGCAGGAAGATATTG	-
y3576-y3577	ATGAGCATGGTGGTGGTCGGCAGCGGCGGCTCTGCGAATCTGAGCATGAGGTGTAGGCTGGAGCTGCTTC	TGTGGAGAATAAACAGCGGG	-
	TTATGTATAAAGACTATCGTCGTAAGGCGATTCAGCCAGAATTTTTTGAACATATGAATATCCTCCTTAG	GTTGAACTCGACGGCGATAT	-
y3641	ATGATTCATAAATTACATCAATATCACCTGGTACGTAGGTTGCGTCAGCAGTGTAGGCTGGAGCTGCTTC	GATCGCTCGTTCAGTTCAGT	-
	TTAATCGCTATACATCAAGTCAATTTCATTCTGATAGCGTTGAAGAATGACATATGAATATCCTCCTTAG	TGGTCACGCGCTGAAATTAC	-
y3654	ATGGAAGCCTATTTATTACATTTATTGACGCAATCCCTGGCTTTCACGCTGTGTAGGCTGGAGCTGCTTC	AGAATTCGACTTCGGTGCCT	-
	TTACCCAGCCAAGACTTTCCACAACAGATGGCGATAGATTGGCATCAGTGCATATGAATATCCTCCTTAG	ATCAGGTGTGCATAAACCGC	-
y3707	ATGAACACCATCAAGTTGGCGAATCCTGGCCCATTGGGCCTTATGGGCTTGTGTAGGCTGGAGCTGCTTC	TTGCTGACGTATGTATCCGC	-
	TTACATTACAGCTTGGGTCTCTAAGGTTTGGCTCGGTTCACCAATAGGCACATATGAATATCCTCCTTAG	ATCACTATCGAGCCGATGAG	-
y3755	GTGATATATGATATTGGTTATGATTATCATTATCATTTGGGTGGGCATCAGTGTAGGCTGGAGCTGCTTC	GATCGTAGGAGTGGCTATTC	-
	TTATCCCCGATGATCGGATACCCCCATCATGACACACCACCGTGCATTAGCATATGAATATCCTCCTTAG	AAGTCTCTAGAATTCGCCCC	-
y3777-y3782	ATGATGAAAGAGCTGATAAATATACTTAAAAATACGCGCCAACATCTGATGTGTAGGCTGGAGCTGCTTC	ATTGCCGGGAAGTTGAGATG	-
	ATGCCGATTCCTGAGCGTTTAACACCAGGCAAAGCCACTAAAAATCGGACCATATGAATATCCTCCTTAG	ATCGCAGAGGAAGTACATCG	-
y3891	TTGGCTTATTTGTTGGTTTTTGATTACAATCGTGAGCGAAAACGAACATTGTGTAGGCTGGAGCTGCTTC	CCCTCTAGATTCAGGGTTTC	-
	TTAAGAAACCAGCGGCAGCGCCTGTTGTTTTTCAGTGTGCGCATCAGCCACATATGAATATCCTCCTTAG	TTTCTCTTCAGACCGGACGA	-
y3927- y3931	ATGCTGATCCAAGGTATTGGCTTGGCTCTCTATCTGGTGGTGGTGCTAATGTGTAGGCTGGAGCTGCTTC	GAGTCGTTGCCGGAAAACAC	-
	GTGACTGGCCCCCCGCTGCGGGTACGCGGTAATGAGGCATTACTGGATGACATATGAATATCCTCCTTAG	TCAGGTTAGCCAGTGCTTCT	-
y3950	ATGAGCACGTTTAAAATAACCATTATTGGCGGTGGGAGTAGCTATACCCCGTGTAGGCTGGAGCTGCTTC	CGGTAGCCATTTTTGCGAGA	-
	TCACAAACCGGAGAACTGAGGCAGGTAAGGTTTATTCACCTGTAGTACCTCATATGAATATCCTCCTTAG	GACGTCTTCCAGTGTAACCA	-
y4034-y4038	TTGAAGGAAATTGCCGTGACGACTCAAAACAGATTCCGAGATAATGAGATGTGTAGGCTGGAGCTGCTTC	CCAGCGCGCAATTTTGAGTT	-
	TTAAATTGCGGGTGTAAGATGGCGATCAGGGGCCTGGCACCAGTTATTTTCATATGAATATCCTCCTTAG	CTGCTTGGCTGAGCTAAACT	-
y4043-y4046	ATGCGATTACGTTTAGCTTTATTTTCGTCATTACTGGCGGCAACCTTTGCGTGTAGGCTGGAGCTGCTTC	TAATCCTGCTCTGGCGCTGC	-
	TTAGCGATAATAAACCGCGATATGCTGGCCTTGTACTTTTTCTATGCTTACATATGAATATCCTCCTTAG	CCGAATATGGGGCTGACCAG	-
y4045-y4047	ATGCATTACAGCTTATTGGTAATACTTGCTGTGGGTTATCGGGTTCGTTCGTGTAGGCTGGAGCTGCTTC	GCATGGGAGCAACATAGAAG	-
	TTGCCAGCAAAGCGGTTATTGCTACTGAGCATCATCGCTCTCGGTGCGATCATATGAATATCCTCCTTAG	TATGGCGATGGGCGATAATC	-
y4050	TTGGCTATTTCCACCGTACTGATGCTCAAACGCGTCTCTTGCCTCGTTCTGTGTAGGCTGGAGCTGCTTC	CCGCAGAAGAAAACCAGAGA	-
	TTAACTGGCGCGCGTTCGCGGCGGGATCAGTGACTTGATTTTCTTGCGCCCATATGAATATCCTCCTTAG	GGATCATCGCTATAGTCTCC	-
y4063	TTGTACTCATTATTAATAATTCTATTATGTGATTGGTATTTCAGAGATATGTGTAGGCTGGAGCTGCTTC	GCCTGAATTCTGCCCATCAA	-
	TTATTTATAATTCAGAGCATAGGTTGCCTGAGAGACAACAGTACCGGTAGCATATGAATATCCTCCTTAG	GAGGGGTGTAATCATCAACC	-
y4067	ATGCAAACTTCATTTTCACCCGCGACACGCTTAGGTCGACGGGCGCTTTTGTGTAGGCTGGAGCTGCTTC	GCTTTTGACATCAACAGCCC	-
	TTACTCGGTAGCAACCGCCTCGGGTTGGCGGCGGATGAACACCATCACCACATATGAATATCCTCCTTAG	CAGCCATTTTGTCACCTTGC	-
y4100-y4101	GTGGCTACCTATTATCTCGCTTCAAAGAAGGAGTTAACGTATATGCGTAAGTGTAGGCTGGAGCTGCTTC	CCCGAAAGTTTGAAACCACG	-
	CTACAACGTCCAGAACAATACCGCCAATATCTGTGGTGACATGATTCGTACATATGAATATCCTCCTTAG	CTATGACTGGCCACATACTC	-
y3066-y3067 (*sucB-sucA*)	ATGCAGAACGGCGCAATGAAGGCCTGGCTGGATTCCTCCTATCTGGCGGGGTGTAGGCTGGAGCTGCTTC	GCCGGCATAATGTCTGTTTC	-
	TTTATACATCAAGTAGCAAGCGAGCAGGATCTTCCAGCATCTCTTTTACCCATATGAATATCCTCCTTAG	CATGCCATACCGAGCAAACA	-
y1173 (*lipB*)	ATGATGCCTCGCTTGCAACAACACAAGATCATTTTACGTCAGCTAGGGTTGTGTAGGCTGGAGCTGCTTC	GGTCAAGATGGTGCTCTAGT	CCATTCACAACGCGCTAATG
	TTACAACACGCTTGATGATGAATGGCTTGATAATAAATAGTCTGATAATGCATATGAATATCCTCCTTAG	CGCCATTTTGTCTGCATCAC	GAGCGCAAAGACTGACTCTT

y1171 (*lipA*)	ATGAGTAAACCGATTCAGATGGAACGCGGCGTAAAATACCGTGATGCAGAGTGTAGGCTGGAGCTGCTTC	CGCCAAAAGTGAATGGCTGA	CCATTCACAACGCGCTAATG
	TTACTTCACTTCCATACCTTTCGCTTGCAGGTCTGCATGGTAAGAAGAGCCATATGAATATCCTCCTTAG	TTTTGCCTGTGCCATATCGC	GAGCGCAAAGACTGACTCTT
y1926 (*lplA)*	ATGTCATCCCTTCGTTTACTCATTTCTGATTCTTATGATCCTTGGTTTAAGTGTAGGCTGGAGCTGCTTC	CGCTCGTCATTGGAGAATGT	CGCTCGTCATTGGAGAATGT
	TTAGCGTAATACTTCTACCAGCCAATGGCAGGCTTGCTGTAACTCGGTTTCATATGAATATCCTCCTTAG	TAGGTTGCAAGCGGGTGTAA	CCAGAGGTTTGCTGTGATAC
y3066-y3067 (*sucB-sucA*)	ATGCAGAACGGCGCAATGAAGGCCTGGCTGGATTCCTCCTATCTGGCGGGGTGTAGGCTGGAGCTGCTTC	GCCGGCATAATGTCTGTTTC	-
	TTTATACATCAAGTAGCAAGCGAGCAGGATCTTCCAGCATCTCTTTTACCCATATGAATATCCTCCTTAG	CATGCCATACCGAGCAAACA	-
PychJ	AGATTTCACTTATCTGGTTGGCCTGCAAGGCCTTCGCGAGGTACCAAAGGTTAAACGGCACCTAA	-	-
	GGATCCGTCGACCTGCAGGTCGACTCTAGAGGTTACCTAGGTACCATAATCAGCAAGGCCACTCG	-	-
PyjeH	AGATTTCACTTATCTGGTTGGCCTGCAAGGCCTTCGCGAGGTACCTGATGGATGAGCAGCAACTG	-	-
	GGATCCGTCGACCTGCAGGTCGACTCTAGAGGTTACCTAGGTACCGCTCTGGCGCTGACTTAG	-	-
PnlpD	AGATTTCACTTATCTGGTTGGCCTGCAAGGCCTTCGCGAGGTACCGTCTTAGAGATCGGAACGGG	-	-
	GGATCCGTCGACCTGCAGGTCGACTCTAGAGGTTACCTAGGTACCCCCATGACAGCAATAATAAACG	-	-
PglnB	AGATTTCACTTATCTGGTTGGCCTGCAAGGCCTTCGCGAGGTACCATCGAATAAACGCCGCAGTA	-	-
	GGATCCGTCGACCTGCAGGTCGACTCTAGAGGTTACCTAGGTACCTACCAATTCTTGCGGCCAAA	-	-
PpldB	AGATTTCACTTATCTGGTTGGCCTGCAAGGCCTTCGCGAGGTACCATGCAGGTCAAGCGACCC	-	-
	GGATCCGTCGACCTGCAGGTCGACTCTAGAGGTTACCTAGGTACCAGTTCAGTTATGTAGCTCTGGTT	-	-
LipA_Ec	ATGAGTAAACCCATTGTGATGGAACGCGGTGTTAAATACCGCGATGCCGAGTGTAGGCTGGAGCTGCTTC	-	-
	TTACTTAACTTCCATCCCTTTCGCCTGCAAATCGGCGTGGTAAGAAGAGCCATATGAATATCCTCCTTAG	-	-
LipB_Ec	TTGTATCAGGATAAAATTCTTGTCCGCCAGCTCGGTCTTCAGCCTTACGAGTGTAGGCTGGAGCTGCTTC	-	-
	TTAAGCGGTAATATATTCGAAGTCCGGATTGTTTAGTAGCGCTAAAATATCATATGAATATCCTCCTTAG	-	-
